# Carbon dioxide equivalent emissions from corn silage fermentation

**DOI:** 10.3389/fmicb.2022.1092315

**Published:** 2023-01-09

**Authors:** Lucas A. Krueger, Lucas R. Koester, David F. Jones, David A. Spangler

**Affiliations:** Department of Research, Development, and Biotechnology, Agri-King, Inc., Fulton, IL, United States

**Keywords:** silage, greenhouse gases, fermentation, carbon dioxide equivalent emissions, sustainability, carbonic anhydrase, lactic acid bacteria

## Abstract

The European Climate Law recently codified the goal for European climate neutrality by 2050, highlighting the need for sustainable farming practices within a robust and transparent carbon dioxide equivalent (CO_2_e) accounting system. In the present study, a series of equations were proposed for the estimation of CO_2_e emissions from corn silage fermentation. Systematic review of previous meta-analyses of corn silage fermentation identified the mean and standard deviation statistics for key model inputs of acetic acid, ethanol, lactic acid, ammonia, and volatile-corrected dry matter loss. Estimates of CO_2_e emissions were determined for a mock dataset comprising 1,000 iterations of randomly-generated values for each metric in accordance with mean and variance statistics of the source data. Estimates for CO_2_e emissions of corn silage based on meta-analysis review of laboratory experiments were 1.9 ± 5.6% (GWP_20_) and 0.2 ± 5.5% (GWP_100_) of silage dry matter. Furthermore, model results demonstrated a precedent for CO_2_ recycling by silage microorganisms, which was supported by genome annotation of strains belonging to common silage species. Linear model equations for GWP_20_ and GWP_100_ with inputs and outputs in mg kg^−1^ silage dry matter were developed, where inputs are acetic acid (A), ethanol (E), lactic acid (L), and volatile corrected dry matter loss (D_V_). Linear equations are (for GWP_20_; Eq. 11):

$${\mathrm{GWP}}_{20}=-3626.1-0.04343A+0.8011E-0.03173L+1.46573D_{V}$$

and for GWP_100_; Eq. 12:

$${\mathrm{GWP}}_{100}=-8526.10-0.22403A-0.11963E-0.03173L+1.46573D_{V.}$$

## 1. Introduction

Recent passage of European Climate Law EU 2021/1119 (European Parliament and the Council of the European Union, 2021) codified the goal for European climate neutrality by 2050 and targeted a 55% reduction in net greenhouse gas emissions from 1990 levels by 2030. Accordingly, national emissions inventories are submitted by participating EU member nations, as well as the United States, to the United Nations Framework Convention on Climate Change. A major focus of such inventories is the emission of carbon dioxide equivalent (CO_2_e) greenhouse gases. As defined in the U.S. 40 CFR Part 98, Equation A-1, CO_2_e is the number of metric tons of CO_2_ emissions with the same global warming potential (GWP) as one metric ton of another greenhouse gas. Pursuant to the European Climate Law and the IPCC Guidelines for National Greenhouse Gas Inventories (Buendia et al., 2019), the agriculture, forestry, and land use sector is noted for its important contribution of CO_2_ sinks that are essential to the transition to climate neutrality. COM(2020)381 (2020) referred to as the EU Farm to Fork Strategy, describes the incentivization of sustainable farming practices within this important sector, especially those which abide within a robust and transparent carbon accounting system. Undoubtedly, land use and conservation are and will remain under pressure to maximize food and feed yields with tandem adoption of sustainable farming practices.

Methods of silage preservation have not yet been considered for their potential impact on sustainability. Optimal silage preservation improves efficiency of land use, conservation of natural resources by preserving nutrient yields, and farmer profitability, but dry matter losses during ensiling can readily exceed 7% of dry matter mass (Rota et al., 2012). As will be demonstrated in this review, optimal silage preservation further decreases direct CO_2_ and CO_2_e emissions to the environment. However, silage preservation is poorly represented in policy-based discussion of sustainable farming practices in deference to pesticide and fertilizer use and efficiency of animal production (COM(2020)381, 2020).

Where international policy encourages farmers to seek out and adopt sustainable farming practices, emissions originating from silage preservation do not have sufficient documentation in the European Union or United States emissions inventories to encourage adoption of good silage preservation practices. In fact, emissions from stored silage are excluded from the European inventory, (European Environment Agency, 2019) whereas many of the factors relating to silage production are inventoried in non-agricultural sectors. For example, although fertilizer use is inventoried with agricultural emissions in the “managed soils” category, agricultural fuel use is inventoried with the energy sector, and cropland is inventoried with the land use, land use change, and forestry (LULUCF) sector. Moreover, the Farm to Fork Strategy (COM(2020)381, 2020) highlighted agricultural emissions as 10.3% of European emissions, but named only improved animal production efficiency, decreased pesticide use, and decreased fertilizer use as key opportunities for ensuring sustainable food production, completely overlooking sustainable silage preservation practices as a viable means to reduce CO_2_e emissions.

Although the present work is focused solely on emissions from silage fermentation, the outcomes are relevant to broader carbon footprint analysis efforts at the level of animal production and farm operations. Quantitative sustainability and CO_2_e footprint efforts have been advancing for well over a decade in dairy (Del Prado et al., 2011; Adom et al., 2012; Henriksson et al., 2014) and beef production (Rotz et al., 2013). Holistic farm carbon footprint assessments have not been limited to feedstuffs, but have also considered the roles of feeds, nutrients, and additives on enteric methane emissions (Little et al., 2017; Meller et al., 2019; Bannink et al., 2020). Furthermore, life cycle and carbon footprint assessments of food products consider not only the mass of product produced, but the nutritional value that is produced (Lee et al., 2021).

Recently, the Global Feed LCA Institute (GFLI) developed a leading database of animal feed life-cycle assessments (Martin et al., 2017). The GFLI database adheres to globally accepted FAO and EU standards, especially FAO LEAP guidelines (FAO, 2016; FAO, 2020) and Feed PEFCR (European Commission, 2018). Although the scope of this work comprises neither a life cycle assessment of silage nor a holistic approach to farm carbon footprint modelling, results are anticipated to quantify CO_2_e emissions from silage that have not been considered previously and that will be made transparent for inclusion in CO_2_e accounting efforts. In this light, the documentation of silage emissions will enable the testing, validation, and adoption of emissions technologies, thereby creating new value to farmers and the food supply chain.

This study does not aim to quantify the effects of silage preservation methods on emissions, but rather, through systematic review and extension of previous meta-analyses, aims to define a standard by which silage emissions can be estimated. This aim specifically encompasses the derivation of an emissions model from what is known about silage fermentation and sets a precedent for inorganic carbon recycling in well-preserved silage. With such a standard in place, it is expected that methods resulting in more efficient silage preservation, lower direct emissions to the environment, and greater farmer profitability can be further recognized as robust and transparent CO_2_e emissions reducing technologies. The emissions model described in following sections considers that dry matter loss from silage is the most direct estimate of total emissions by mass, but not all emissions are comprised of CO_2_. Rather, dry matter loss is considered to encompass three critical fractions: (1) direct CO_2_ emissions from fermentation metabolism, (2) evaporation of non-CO_2_ volatile organic compounds (VOC), and (3) CO_2_ and metabolic water from aerobic respiration. These are equated to CO_2_e emissions in terms C_M_, C_O_, and C_R_ in further sections.

## 2. Materials and methods

### 2.1. Statistical analysis

All statistical procedures were carried out using Statistix 10 (Analytical Software, Tallahasse, FL). Linear model terms and differences among means were significant where *p* < 0.05.

### 2.2. Estimating CO_2_e emissions from silage

A series of equations is proposed to account for CO_2_e emissions from silage preservation. In the equations that follow, all terms are scaled to units of mg kg^−1^ (ppm) values. Terms include concentration of lactic acid, acetic acid, ethanol, ammonia, dry matter loss, volatile-corrected dry matter loss, and the concentration of all minor VOC. Through systematic review and extension of data, the three fractions of dry matter loss stated above are equated to CO_2_e emissions. Methods stated in this section include minimal discussion of assumptions, with these concepts elaborated thoroughly in Discussion.

#### 2.2.1. Direct CO_2_ emissions from fermentation

The first fraction of dry matter loss to be equated to CO_2_e emissions is direct CO_2_ emissions from fermentation metabolisms (C_M_). Direct CO_2_ emissions from fermentation specifically comprise CO_2_ produced from the decarboxylation of pyruvate in fermentation pathways. Fermentation products in silage are known to include lactic acid, acetic acid, ethanol, and numerous other VOC. A previous meta-analysis (Hafner et al., 2013) documented the mean concentration of 32 VOC in silage. This mean reported concentration (MRC) is re-iterated in Table 1. Also stated in Table 1 is the molar CO_2_ equivalent (MCE), which is the stoichiometric ratio of mol CO_2_ produced per mol of each fermentation product from a hexose sugar. Direct metabolic production of CO_2_ (calculated direct metabolic production of CO_2_ in mg kg^−1^; CDMP) for each VOC is calculated through Eq. 1:


(1) $$\mathrm{CDMP}=\mathrm{MRC}\times {\left(\mathrm{molar mass}\right)}^{-1}\times \mathrm{MCE}\times 44.01$$

where variables MRC (mg kg^−1^), molar mass, and MCE for each compound are presented in Table 1, and constant 44.01 is the molar mass of CO_2_. Therefore, CDMP (Table 1) is the mass of CO_2_ released during the metabolism of a hexose sugar to each respective VOC.

**Table 1:** Volatile organic compounds in silage and molar equivalent CO_2_ release for metabolite production.

Compound name	CAS number	Molar mass, g mol^−1^	MCE^1^	MRC^2^ mg kg^−1^	MRC mmol kg^−1^	Pyruvate^3^ mmol kg^−1^	CDMP^4^	EBIR^5^	GWP_20_^6^ from O_3_	GWP_100_^6^ from O_3_
Acetic acid	64-19-7	60.05	1	1.3 × 10^4^	2.1 × 10^2^	2.1 × 10^2^	9.2 × 10^3^	0.20	5.3 × 10^3^	1.1 × 10^3^
Propionic acid	79-09-4	74.08	0	1.6 × 10^3^	2.1 × 10^1^	2.1 × 10^1^		0.34	1.1 × 10^3^	2.2 × 10^2^
Butyric acid	107-92-6	88.11	2	1.6 × 10^2^	1.8 × 10^0^	3.6 × 10^0^	1.6 × 10^2^	0.55	1.8 × 10^3^	3.6 × 10^2^
Isobutyric acid	79-31-2	88.11	0	3.2 × 10^2^	3.6 × 10^0^			0.38	2.5 × 10^2^	5.0 × 10^1^
Isovaleric acid	503-74-2	102.13	0	2.5 × 10^0^	2.5 × 10^−2^			0.96	4.9 × 10^0^	9.8 × 10^−1^
Methanol	67-56-1	32.04	0	2.0 × 10^2^	6.2 × 10^0^			0.20	8.2 × 10^−1^	1.6 × 10^−1^
Ethanol	64-17-5	46.07	1	5.0 × 10^3^	1.1 × 10^2^	1.1 × 10^2^	4.8 × 10^3^	0.57	5.8 × 10^3^	1.2 × 10^3^
1-Propanol	71-23-8	60.10	0	1.0 × 10^3^	1.7 × 10^1^	1.7 × 10^1^		0.79	1.6 × 10^3^	3.2 × 10^2^
2-Propanol	67-63-0	60.10	0	1.6 × 10^2^	2.6 × 10^0^	2.6 × 10^0^		0.26	8.5 × 10^1^	1.7 × 10^1^
2-Propenol	107-18-6	58.10	0	7.9 × 10^0^	1.4 × 10^−1^	1.4 × 10^−1^		2.75	4.4 × 10^1^	8.9 × 10^0^
2-Methyl-1-propanol	78-83-1	74.12	0	1.0 × 10^1^	1.4 × 10^−1^	1.4 × 10^−1^		0.72	1.5 × 10^1^	3.0 × 10^0^
1-Butanol	71-36-3	74.12	2	6.3 × 10^0^	8.5 × 10^−2^	1.7 × 10^−1^	7.5 × 10^0^	0.88	1.1 × 10^1^	2.3 × 10^0^
2-Butanol	78-92-2	74.12	2	1.0 × 10^2^	1.4 × 10^0^	2.7 × 10^0^	1.2 × 10^2^	0.50	1.0 × 10^2^	2.1 × 10^1^
3-Methyl-1-butanol	123-51-3	88.15	1	1.6 × 10^1^	1.8 × 10^−1^	3.6 × 10^−1^	7.9 × 10^0^	0.90	2.9 × 10^1^	5.9 × 10^0^
1-Hexanol	111-27-3	102.16	0	1.0 × 10^1^	9.8 × 10^−2^			0.82	1.7 × 10^1^	3.4 × 10^0^
2-Phenylethanol	60-12-8	122.17	1	1.0 × 10^1^	8.2 × 10^−2^	8.2 × 10^−2^	3.6 × 10^0^	1.07	2.2 × 10^1^	4.4 × 10^0^
Acetone	67-64-1	58.08	0	1.3 × 10^1^	2.2 × 10^−1^	2.2 × 10^−1^		0.09	2.4 × 10^0^	4.8 × 10^−1^
2-Butanone	78-93-3	72.11	2	2.5 × 10^1^	3.5 × 10^−1^	7.0 × 10^−1^	3.1 × 10^1^	0.37	1.9 × 10^1^	3.8 × 10^0^
3-Hydroxy-2-butanone	513-86-0	88.11	1	1.1 × 10^3^	1.3 × 10^1^	2.6 × 10^1^	5.6 × 10^2^	0.37	8.3 × 10^2^	1.7 × 10^2^
Methyl acetate	79-20-9	74.08	1	2.5 × 10^2^	3.4 × 10^0^	3.4 × 10^0^	1.5 × 10^2^	0.04	2.0 × 10^1^	4.1 × 10^0^
Ethyl acetate	141-78-6	88.11	2	1.3 × 10^2^	1.4 × 10^0^	2.9 × 10^0^	1.3 × 10^2^	0.24	6.4 × 10^1^	1.3 × 10^1^
Ethyl lactate	97-64-3	118.13	1	2.0 × 10^2^	1.7 × 10^0^	3.4 × 10^0^	7.4 × 10^1^	0.59	2.4 × 10^2^	4.8 × 10^1^
Propyl acetate	109-60-4	102.13	1	2.5 × 10^2^	2.5 × 10^0^	4.9 × 10^0^	1.1 × 10^2^	0.31	1.6 × 10^2^	3.2 × 10^1^
Propyl lactate	616-09-1	132.16	0	1.6 × 10^2^	1.2 × 10^0^	2.4 × 10^0^		0.35	1.1 × 10^2^	2.3 × 10^1^
Acetaldehyde	75-07-0	44.05	1	1.0 × 10^2^	2.3 × 10^0^	2.3 × 10^0^	2.0 × 10^2^	1.61	3.3 × 10^2^	6.6 × 10^1^
Propionaldehyde	123-38-6	58.08	0	1.3 × 10^1^	2.2 × 10^−1^	2.2 × 10^−1^		1.71	4.5 × 10^1^	9.1 × 10^0^
2-Methylpropanal	78-84-2	72.11	0	1.6 × 10^1^	2.2 × 10^−1^	2.2 × 10^−1^		1.35	4.4 × 10^1^	8.9 × 10^0^
Butyraldehyde	123-72-8	72.11	2	1.6 × 10^1^	2.2 × 10^−1^	4.4 × 10^−1^	1.9 × 10^1^	1.45	4.7 × 10^1^	9.5 × 10^0^
3-Methylbutanal	590-86-3	86.13	1	1.0 × 10^2^	1.2 × 10^0^	2.3 × 10^0^	5.1 × 10^1^	1.21	2.5 × 10^2^	5.0 × 10^1^
Valeraldehyde	110-62-3	86.13	2	1.0 × 10^2^	1.2 × 10^0^	2.3 × 10^0^	1.0 × 10^2^	1.26	2.6 × 10^2^	5.2 × 10^1^
Hexanal	66-25-1	100.17	0	1.0 × 10^2^	1.0 × 10^0^			1.07	2.2 × 10^2^	4.4 × 10^1^
Heptanal	111-71-7	114.19	1	1.3 × 10^1^	1.1 × 10^−1^	2.2 × 10^−1^	4.9 × 10^0^	0.90	2.4 × 10^3^	4.8 × 10^2^
All VOC				**2.4 × 10**^4^	**4.0 × 10**^2^	**4.2 × 10**^2^	**1.6 × 10**^4^	**0.35**	**1.7 × 10**^4^	**3.5 × 10**^3^
Minor VOC				**6.2 × 10**^3^	**8.4 × 10**^1^	**9.9 × 10**^1^	**1.6 × 10**^3^	**0.49**	**6.2 × 10**^3^	**1.3 × 10**^3^

Bold, summary of values of all VOC or minor VOC as utilized in text. ^1^Metabolic CO_2_ equivalent for mol CO_2_ produced metabolically per mol of each metabolite produced. ^2^Mean reported concentrated in silage, mg kg^−1^ on a dry matter basis Hafner et al., 2013. ^3^Pyruvate, mmol kg^−1^ silage dry matter produced as intermediate metabolite for each VOC at given MRC.^4^Calculated direct metabolic CO_2_ production, mg kg^−1^ in silage on a dry matter basis, resulting from metabolic pathway to respective metabolite at MRC. ^5^Equal benefit incremental reactivity, g O_3_ per g VOC Hafner et al., 2013. ^6^Partial global warming potential for tropospheric O_3_ produced from silage VOC at MRC, carbon-equivalents as mg kg^−1^ in silage on a dry matter basis.

In equations that follow, hexoses comprise 65% of fermentable sugars and pentoses comprise the remaining 35%. The basis for this assumption is an *ad hoc* analysis of sugars in fresh, green chop corn silage samples (*n* = 6) conducted by Analab (Agri-King, Inc., Fulton, IL 61252) according to AOAC method 2018.16. Pentose sugars comprised 35.0 ± 9.6% (mean ± SD) of available sugars by mass.

Eq. 2 estimates total metabolic production of CO_2_ from the fermentation of sugars to acetic acid, ethanol, and minor VOC. Acetic acid and ethanol are common measures in silage, whereas other minor VOC are not. Therefore, concentration of acetic acid and ethanol are included as variables, whereas concentration of minor VOC is a constant. Table 1 documents that the minor VOC account for approximately 1.6 × 10^3^ mg CO_2_ kg^−1^ silage dry matter. Where acetic acid and ethanol are quantified per kg silage dry matter and minor VOC are estimated at MRC, direct CO_2_ emission from metabolite production (C_M_) is calculated by:


(2) $$C_{M}=0.65\times \left[\left(A\frac{M_{C}}{M_{A}}\right)+\left(E\frac{M_{C}}{M_{E}}\right)+\left(1.6\times 10^{3}\right)\right]$$

where C_M_ is mg CO_2_, A is mg acetic acid, E is mg ethanol, M_A_ is the molar mass of acetic acid (60.05 g mol^−1^), M_E_ is the molar mass of ethanol (46.07 g mol^−1^), M_C_ is the molar mass of carbon dioxide (44.01 g mol^−1^), constant 1.6 × 10^3^ is the contribution from minor VOC at MRC, and constant 0.65 represents the portion of hexoses among total sugars from which acetate, ethanol, and minor VOC are derived.

#### 2.2.2. Evaporation of non-CO_2_ volatile organic compounds

Estimates for CO_2_e emissions of non-CO_2_ VOC from silage dry matter comprise the evaporation of acetic acid, ethanol, and the minor VOC coupled with propensity to form low atmospheric (tropospheric) O_3_. These estimates are scaled as global warming potential (GWP) in CO_2_e equivalents on a mg kg^−1^ basis in silage dry matter on 20 year and 100 year horizons and are termed C_O-20_ and C_O-100_ (Terms named GWP_20_ and GWP_100_ are reserved for cumulative estimates of CO_2_e emissions).

Evaporation of VOC occurs at ambient temperature over a duration of many hours as silage faces are exposed and silage is further defaced, mixed into feed, and delivered into the feedbunk (Hafner et al., 2013; Robinson et al., 2016). Volatility coefficients of 0.554 and 0.991 (Porter and Murray, 2001) are applied to the concentrations of acetic acid and ethanol, which are variable in Eqs 3 and 4 below. Concentration of minor VOC is assumed constant at MRC, which was reported by Hafner et al. (2013). Estimated O_3_ formation from emitted VOC is listed as equal benefit incremental reactivity (EBIR) in Table 1 and also is adapted from Hafner et al. (2013). These data are extended in Table 1 to provide weighted mean EBIR for the minor VOC of 0.49.

The CO_2_e of O_3_ is stated as 2.04 on a 20 year horizon and 0.41 on a 100 year horizon. These values are derived as an extension of data reported by Ramaswamy et al. (2001), from an estimate of relative normalized radiative forcing (681.0; Table 2) amortized to the atmospheric lifetime of O_3_. This estimate is elaborated in Discussion. Constants in Eq. 3 and Eq. 4 of 6.2 × 10^3^ and 1.3 × 10^3^, respectively, describe the CO_2_e of the minor VOC based on concentration, EBIR, and GWP of O_3_. These constants are stated in Table 1. Therefore, the CO_2_e emissions of VOC through O_3_ formation are represented on 20 and 100 year horizons as C_O20_ and C_O100_ in Eq. 3 and Eq. 4, respectively.


(3) $$C_{O20}=\left(6.2\times 10^{3}\right)+2.04\times \left[\begin{array}{l} \left(A\times 0.554\times {\mathrm{EBIR}}_{A}\right)+\\ \left(E\times 0.991\times {\mathrm{EBIR}}_{E}\right) \end{array}\right]$$

(4) $$C_{O100}=\left(1.3\times 10^{3}\right)+0.41\times \left[\begin{array}{l} \left(A\times 0.554\times {\mathrm{EBIR}}_{A}\right)+\\ \left(E\times 0.991\times {\mathrm{EBIR}}_{E}\right) \end{array}\right]$$

**Table 2:** Radiative forcing normalized to abundance of atmospheric compounds.

Compound	Abundance over pre-industrial level, ppm^1,2^	Radiative forcing from pre-industrial level, Wm^−2^	Radiative forcing normalized to abundance	RNRF^3^
CO_2_	87	+1.46^1^	0.0168	1.0
CH_4_	1.045	+0.48^1^	0.4593	27.4
O_3_	0.035	+0.40^2^	11.429	681.0

^1^Ramaswamy et al., 2001. ^2^Brasseur et al., 2001. ^3^Relative normalized radiative forcing in CO_2_-equivalents for the estimated increase in radiative forcing from pre-industrial levels per the increase in abundance over pre-industrial levels.

#### 2.2.3. CO_2_ from respiration

Respired CO_2_ is estimated from empirically measured dry matter loss after subtracting mass lost through glycolysis and the evaporation of VOC. Respired mass is converted to mass of CO_2_ through molar ratios.

Eq. 5 determines mass lost to the production of pyruvate through glycolysis. Eq. 5 sums the molar concentration of pyruvate required for the manufacture of lactic acid (L), acetic acid (A), ethanol (E), and minor VOC (constant at 99.2 mmol kg^−1^). In Eq. 5, L, A, and E are in units of mg kg^−1^ and M_L_, M_A,_ and M_E_ the respective molecular weights for each compound. Mass losses from glycolysis to pyruvate are 3 g mol^−1^ and hexose sugars comprise 65% of fermentable sugars.


(5) $$P=0.65\times 3.0\times \left(\frac{L}{M_{L}}+\frac{A}{M_{A}}+\frac{E}{M_{E}}+99.2\right)$$

Eq. 6 determines the mass of volatiles lost during determination of dry matter loss. Volatility coefficients are from Porter and Murray (2001) for drying at 60°C. In addition to variables A, E, and L stated for Eq. 5, ammonia in mg kg^−1^ is input as variable N.


(6) $$V=0.554A+0.991E+1.003N+0.090L+\left(6.2\times 10^{3}\right)$$

Eq. 7 further determines respired CO_2_ as term C_R_ by subtracting terms P (from Eq. 5), C_M_ (from Eq. 2), and V (from Eq. 6) from dry matter loss determined by oven dry matter. This estimate of respired mass is converted through molar ratios where 1 mol glucose is respired to 6 mol CO_2_, constant 44.01 is the molar mass of CO_2_ and constant 180.156 is the molar mass of glucose.


(7) $$C_{R}=6\times 44.01\times \frac{\left(D-P-C_{M}-V\right)}{180.156}$$

Where volatile corrected dry matter loss (vcDML) is considered in later review and discussion, adapted equations include D_V_ to denote vcDML as a substitute for oven dry matter loss (term D).

#### 2.2.4. Modified equation for utilizing vcDML as input

Where vcDML is utilized in lieu of oven dry matter loss in Eq. 7, the equation term V (calculated in Eq. 6) should be equal to 6.2 × 10^3^, to reflect only the evaporation of minor VOC that is assumed to be constant. This is the result of accounting for the evaporation of A, E, L, and N in the determination of vcDML, rather than in Eq. 6. With this modification in practice, vcDML as term D_V_ is substituted for D in Eq. 7. It should again be noted that all equations are scaled to mg kg^−1^ values. This substituted equation is shown as Eq. 8:


(8) $$C_{R}=6\times 44.01\times \frac{\left(D-P-C_{M}-6.2\times 10^{3}\right)}{180.156}$$

#### 2.2.5. Sum of CO_2_e emissions

Where oven dry matter loss, lactic acid, acetic acid, ethanol, and ammonia are measured empirically in silage per kg dry matter, and minor VOC are present at MRC, total CO_2_e emissions are determined by the following equation:


(9) $$CO_{2}e=C_{M}+C_{OT}+C_{R}$$

where C_M_ is direct CO_2_ emissions from the fermentation of sugars to total VOC, C_R_ is direct CO_2_ emissions from respiration, and C_OT_ is either C_O20_ or C_O100_ for O_3_ originating from emitted VOC.

#### 2.2.6. Systematic review of silage fermentation metrics for estimation of GWP

Measures A, E, L, N, and D, which are inputs to Eqs 2 through 8, are known to be variable in fermented silage. Existing meta-analyses were reviewed to define mean and variance estimates for these measures to enable further estimation of mean and variance for GWP of corn silage fermentation.

Five meta-analysis studies were identified in which fermentation outcomes were characterized for untreated whole crop corn (maize) silages. Of these five studies, one analysis (Kasmaei et al., 2013) reviewed observations specifically from the Swedish University of Agricultural Sciences and did not specify observations from studies published in the literature. Three others reviewed the effects of a silage additive. Specifically, Kleinschmit and Kung (2006) reviewed the effects of *Lentilactobacillus buchneri* (formerly *Lactobacillus buchneri;* Zheng et al., 2020) compared with control and this analysis was updated and made more comprehensive by Arriola et al. (2021), whereas Da Silva and Kung (2022) reviewed the effects of a chemical additive compared with control. Lastly, Blajman et al. (2018) reviewed 104 published studies that included a control and were reported between calendar years 1980 and 2017.

Of the meta-analysis studies named, those by Arriola et al. (2021) and Blajman et al. (2018) are noted as the most comprehensive. The report by Blajman et al. (2018) failed to specifically cite each of the 104 works included in the analysis, whereas the meta-analysis by Arriola et al. (2021) provided complete citation of studies (Supplementary Table S1; Arriola et al., 2021). This provision of references enabled further examination of data and methods, which was necessary since this report summarized fermentation outcomes from multiple ensiled forage types (62 studies of corn silage out of 158 studies in the meta-analysis). Because of the comprehensive scope and transparency of data, the values and uncertainties reported by Arriola et al. (2021) were considered to be the best available meta-data to describe the fermentation outcomes of corn silage.

Mean and standard deviation for A, E, L, N, and D were summarized for treatment means of control corn silage samples in the meta-analysis by Arriola et al. (2021). Since not all studies reported each metric, the number of studies and the number of treatment means reported were also summarized.

Data summarized by Arriola et al. (2021) were further extended to estimate vcDML. Studies reviewed by Arriola et al. (2021) were examined to determine if dry matter loss was reported directly as vcDML or as oven dry matter loss, as shown in Table 3. Of the 35 studies that reported a dry matter loss value, 4 studies reported vcDML directly, and another 30 studies reported oven dry matter loss with additional data on the concentration of fermentation products such as lactic acid, acetic acid, ethanol, and ammonia. With the aim of summarizing vcDML from these studies in the meta-analysis, a vcDML value was calculated for each treatment mean using coefficients described by Porter and Murray (2001). Namely, where oven dry matter was determined by drying at 60°C, volatility coefficients for lactic acid, acetic acid, ethanol, and ammonia were 0.090, 0.554, 0.991, and 1.003, respectively. Where oven dry matter was determined by drying at 100°C, the same respective coefficients were 0.375, 0.892, 0.975, and 0.987.

**Table 3:** Evaluation of dry matter loss in studies summarized by Arriola et al. (2021).

	Number of studies	Number of treatment means	Mean	SD	SEM
Dry matter loss reported in publication	35	74	3.6	3.4	0.4
Insufficient data to determine volatile corrected dry matter loss	5	13			
Volatile corrected dry matter loss value obtained (direct reporting or calculated value)	30	61	1.1	3.8	0.5
Volatile corrected dry matter loss reported directly	4	13	3.1	2.0	0.5
Volatile corrected dry matter loss calculated during review	26	48	0.6	4.0	0.6
Calculated with data provided	11	18	0.7	3.1	0.7
Calculated with assumptions	15	30	0.6	4.4	0.8
Calculations using 60°C coefficients for volatile correction	22	39	1.2	3.9	0.6
Calculations using 100°C coefficients for volatile correction	4	9	-1.9	3.3	1.1

Of the 30 studies and 61 treatment means for which a vcDML value was obtained, approximately half (15 studies, 30 treatment means) provided incomplete data on the concentration of fermentation products. Each of these studies did report lactic and acetic acids, each study omitted ammonia as a % of dry matter, and 8 studies omitted ethanol. Where either ammonia, ethanol, or both values were omitted, calculations for vcDML were populated with the mean ethanol or ammonia value reported in Table 4.

**Table 4:** Mean fermentation values and uncertainties in corn silage based on meta-analysis by Arriola et al. (2021).

Metric, % of DM	Mean ± SD	Number of studies	Number of treatment means
Dry matter loss, %	3.6 ± 1.2	35	74
Acetic acid, %	1.6 ± 0.8	60	117
Ethanol, %	1.0 ± 0.8	38	79
Lactic acid, %	5.4 ± 2.2	58	112
Ammonia, %	0.2 ± 0.3	24	51

### 2.3. Estimation of GWP_20_ and GWP_100_ with randomly generated data

With the aim of providing mean and variance estimates for GWP_20_ and GWP_100_ of corn silage fermentation, the mean and standard deviation of A, E, L, N, and D_V_ from Tables 3, 4 guided the generation of random data as a mock dataset with 1,000 iterations of each metric. The mean and SD for each metric determined by Arriola et al. (2021) were used as boundaries in the data generation step and are reiterated in Table 5. Term D was dropped from the analysis in favor of utilizing D_V_ as input for estimates.

**Table 5:** Parameters for generation of random data.

Metric, % of DM	Mean ± SD	Transformation	Transformed Mean ± SD	W^1^
Lactic acid	5.4 ± 2.2	Square root	2.27 ± 0.48	0.986
Acetic acid	1.6 ± 0.8	Box-cox; λ = 0.47	0.44 ± 0.60	0.964
Ammonia	0.2 ± 0.3	Box-cox; λ = −0.21	−2.43 ± 1.26	0.970
Ethanol	1.0 ± 0.8	Box-cox; λ = 0.38	−0.24 ± 0.89	0.977
vcDML	1.1 ± 3.8	Box-cox as vcDML+10; λ = 0.18	2.95 ± 0.51	0.974

^1^Shapiro-Wilk normality test.

All source data for each of the metrics in Table 5 were found to be distributed non-normally by Shapiro–Wilk normality test. Since random generation of data with a normal distribution would therefore be incompatible with the source data, values were transformed using a Box-Cox transformation for all metrics except lactic acid, for which a square root transformation was optimal. Box-Cox optimal λ value for each metric was determined by inputting indexed source data into an online tool.^1^ In order to accomplish the Box-Cox transformation for D_V_, data were first adjusted by +10 to render all values positive. The Box-Cox transformation function for D_V_ was:


(10) $$y=\frac{\left[{\left(D_{V}+10\right)}^{0.18}-1\right]}{0.18}$$

Table 5 further documents the mean and SD of the transformed values. For each metric of A, E, L, N, and D_V_, transformed mean and standard deviation were used as input values for population mean and variance for the generation of random data (1,000 iterations) with normal distribution *via* an online tool.^2^ Randomly generated data were then back-transformed and assembled into a dataset of 1,000 mock silage samples. Then, GWP_20_ and GWP_100_ were calculated for each mock sample according to Eqs 2 through 6 and Eqs. 8 and 9. Therefore, each GWP_20_ and GWP_100_ estimate was produced from data that abided by the population statistics of the original non-normal source data summarized by Arriola et al. (2021). Values for GWP_20_, GWP_100_ and model terms C_M_, C_O20_, C_O100_, and C_R_ were summarized as mean, SD, and SEM for the dataset of 1,000 mock samples (Table 6).

**Table 6:** Estimates of GWP_20_ and GWP_100_ and associated model terms from randomly generated population data.

Metric, % of DM	Mean ± SD	95% Low CI	95% High CI
GWP_20_	1.9 ± 5.6	−9.2	13.1
GWP_100_	0.2 ± 5.5	−10.8	11.2
C_M_	1.5 ± 0.6	0.3	2.7
C_O-20_	2.2 ± 1.1	0.1	4.3
C_O-100_	0.4 ± 0.2	0.0	0.9
C_R_	−1.8 ± 5.5	−11.7	8.1

CI, confidence interval.

### 2.4. Derivation and validation of linear models for GWP_20_ and GWP_100_

The authors assert that Eq. 1 through Eq. 9 are cumbersome and prohibitive to the practical application of GWP estimates for silage fermentation. To simplify the set of equations for practical use, the mock dataset was subject to linear regression to derive coefficients for the direct estimation of GWP_20_ and GWP_100_ from empirical measurements A, E, L, N, and D, using each as an unforced, independent starting variable. It should be again noted that vcDML as term D_V_ was substituted for D in the mock data, such that term *V* (calculated in Eq. 6 and input in Eq. 7) was constant at 6.2 × 10^3^ mg kg^−1^. Because N is only considered in Eq. 2 through Eq. 7 with regard to dry matter loss, and term D_V_ was inputted to the regression, N was already accounted for by term D_V_. Therefore N was not anticipated to be a significant model term, although its determination in silage is essential for calculating D_V_.

Whereas linear model equations were derived from the mock dataset, the model was validated against the original source data. Values for GWP_20_ and GWP_100_ were calculated by using Eqs. 2 through 9 and by using respective linear model equations. Identical values between the two methods revealed, as expected, that the linear model accurately condensed Eqs 2 through 9 and was therefore valid.

### 2.5. Genome annotation of silage microorganisms

As will be presented in further sections, model outcomes indicated a strong likelihood for inorganic carbon recycling during corn silage fermentation. To augment review that is presented below, the authors queried publicly available genomes of four common silage organisms to evaluate the presence of 10 known genes, including aquaporin GlpF, carbonic anhydrase, and several carboxylase enzymes known to incorporate bicarbonate ion into organic biomolecules. Query strains were *Lactiplantibacillus plantarum* SK151, *Levilactobacillus brevis* NPSQW145, *Lentilactobacillus buchneri* ATCC4005 (all formerly classified as *Lactobacillus,* Zheng et al., 2020), and *Lactococcus lactis* LAC640. Open reading frames from each genome were predicted using Prokka v1.14.6 (Seemann, 2014) and annotated using eggNOG-mapper v2 (Cantalapiedra et al., 2021). Gene matches identified in the annotations were confirmed by BLAST search.

### 2.6. Data availability

All silage fermentation profile data used to define model constants were generated or adopted from published sources as cited. All genomes of common silage inoculant bacteria are freely available through the National Center for Biotechnology Information GenBank database. GenBank accession numbers for each genome are provided in Supplementary Table S1.

## 3. Results

Principal findings of the present work comprise estimates of mean and variance statistics for corn silage fermentation outcomes and estimates of GWP_20_ and GWP_100_ based on extension of these primary data.

### 3.1. Corn silage fermentation outcomes

Review and extension of the data reported by Arriola et al. (2021) resulted in mean and variance estimates for measures A, E, L, N, D, (Table 3) and D_V_ (Table 4). These estimates guided the generation of random data for a mock dataset to inform estimates of GWP_20_ and GWP_100_. Specifically, term D_V_ was estimated at 1.1 ± 3.8% of silage dry matter, whereas oven dry matter was reported at 3.6 ± 3.4%. Analysis shown in Table 4 further summarizes this term. Importantly, vcDML for samples dried at 60°C was 1.2 ± 3.9%, but was−1.9 ± 3.3% for samples dried at 100°C. This notable difference was observed despite applying two different sets of volatility coefficients as prescribed by Porter and Murray (2001). The validity and implications of D_V_ < 0 are elaborated in Discussion.

### 3.2. Estimation of GWP_20_ and GWP_100_ with randomly generated data

Estimates for GWP_20_, GWP_100_ and associated model terms C_M_, C_O-20_, C_O-100_, and C_R_ are recorded in Table 6. Notably, Eqs. 1 through 7 operate in units of mg kg^−1^, but outcomes were converted to percentage units for display in Table 6. The GWP_20_ of corn silage fermentation (mean ± SD) was estimated at 1.9 ± 5.6% of silage dry matter, whereas GWP_100_ was estimated at 0.2 ± 5.5%. Respective mean ± SEM values are 1.9 ± 0.2 for GWP_20_ and 0.2 ± 0.2 for GWP_100_. Therefore, GWP_20_ was significantly greater than zero (analyzed by one-sample t-test), whereas GWP_100_ was not significantly different from zero (*p* = 0.267).

Model term C_R_ was estimated at-1.8 ± 5.5% of dry matter (mean ± SD). This value again is significantly differently from zero (mean ± SEM of-1.8 ± 0.2; *p* < 0.001). The validity and implications of D_V_ < 0 and C_R_ < 0 are elaborated in Discussion.

### 3.3. Derivation of linear models for GWP_20_ and GWP_100_

Linear regression procedures produced a simplified linear model equations for GWP_20_ and GWP_100_ to substitute for cumbersome Eqs. 2 through 9. The linear model established by regression for GWP_20_ was:


(11a) $$\begin{array}{l} {\mathrm{GWP}}_{20}=-3626.1-0.04343A+0.80111E-\\ \;\;\;\;\;\;\;\;\;\;\;\;\;\;\;\;\;\;\;\;\;\;\;\;\;\;0.03173L+1.46573D_{V} \end{array}$$

where variables A, E, L, and D_V_ are the concentration of acetic acid, ethanol, lactic acid, and volatile corrected dry matter loss on a dry matter basis, respectively, and all values are expressed in mg kg^−1^. The linear model established by regression for GWP_100_ was:


(12a) $$\begin{array}{l} {\mathrm{GWP}}_{100}=-8526.1-0.22403A-0.11963E-\\ \;\;\;\;\;\;\;\;\;\;\;\;\;\;\;\;\;\;\;\;\;\;\;\;\;0.03173L+1.46573D_{V} \end{array}$$

where variables and model constraints are as described for Eq. 11. Because the linear equations were derived from a set of mathematical equations (Eq. 2 through Eq. 9), model *R*^2^ = 1 and coefficient standard error values were 0.

### 3.4. Genome annotation of silage microorganisms

**Figure 1:**
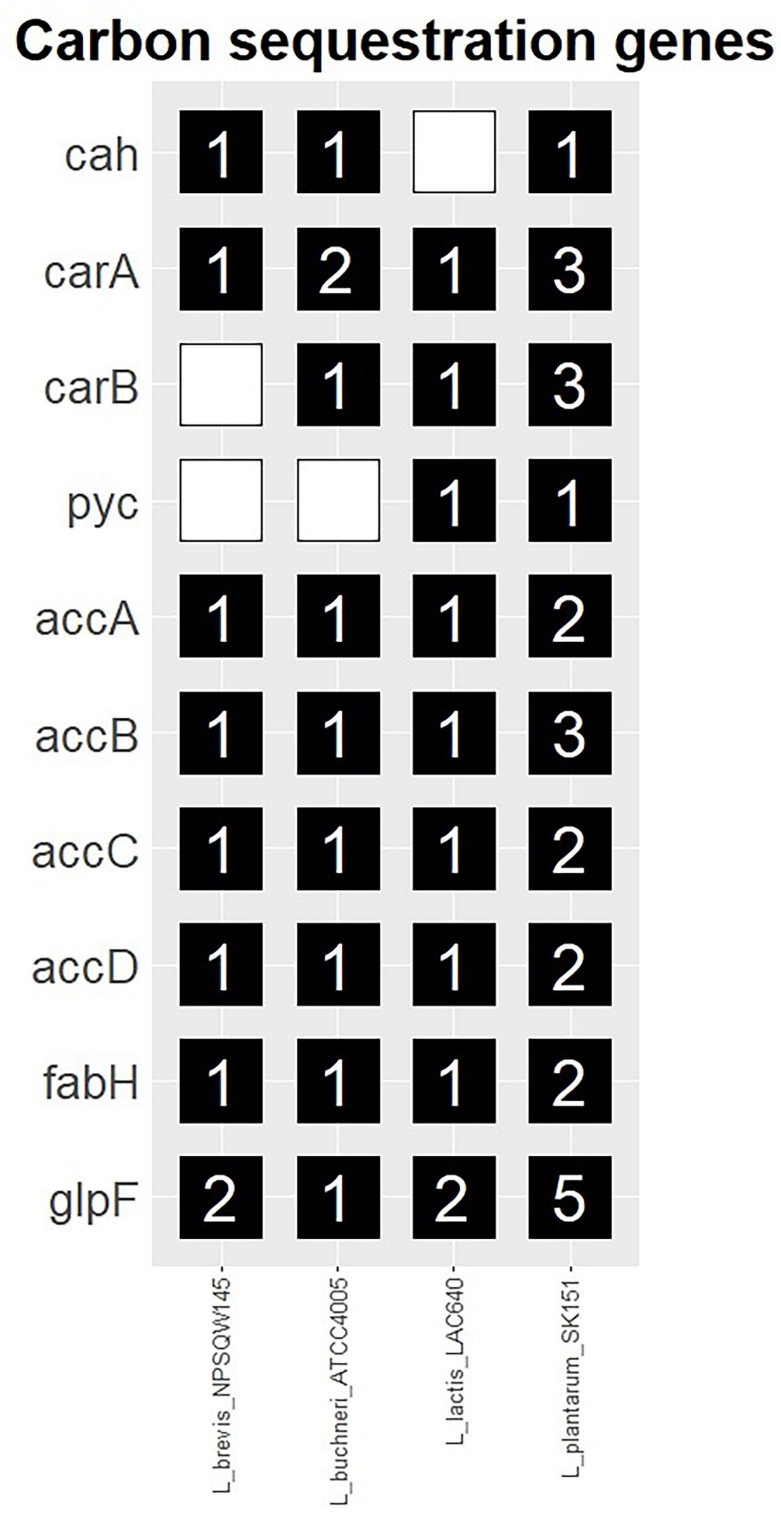
Prevalence of carbonic anhydrase and carboxylase genes, and the aquaglyceroporin glpF identified in common silage species.

Genes of interest for their roles in inorganic carbon assimilation are described in Table 7 and their gene copy number within query genomes is shown in Figure 1. As shown in Figure 1, each of the 10 genes of interest for their roles in inorganic carbon assimilation were identified in one or more of the evaluated strains. At least one copy of both aquaporin glpF was present in all of the strains. Carbonic anhydrase (cah) was present in the *Lactobacillus* and *Lactiplantibacillus* genomes, but not in *Lactococcus lactis*. *Lactiplantibacillus plantarum* was found to harbor all genes of interest and carried a greater number of genes than the other strains. Sequences from query-matched open reading frames are documented in Supplementary Table S2. The results from related BLAST searches are documented in Supplementary Table S3.

**Table 7:** Genes queried in analysis for inorganic carbon assimilation functions in common silage organisms.

Preferred name	Description
cah	Carbonic anhydrase
carA	Carbamoyl-phosphate synthase small chain
carB	Carbamoyl-phosphate synthase large chain
pyc	Pyruvate carboxylase
fabH	Acetyl-coenzyme A carboxylase carboxyl transferase subunit alpha
accB	Biotin carboxyl carrier protein of acetyl-CoA carboxylase
accC	Biotin carboxylase
accD	Acetyl-coenzyme A carboxylase carboxyl transferase subunit beta
accA	3-oxoacyl-[acyl-carrier-protein] synthase 3
glpF	Glycerol facilitator uptake protein

## 4. Discussion

### 4.1. Key model terms

Text provided in Materials and Methods was limited to information that was immediately pertinent to CO_2_e model equations. That information provided sufficient background and logic in most cases, but elaboration on a few critical points is provided here.

#### 4.1.1. GWP of O_3_

The GWP_20_ and GWP_100_ for O_3_ have not been reported previously, so the estimates utilized in model equations comprise a novel extension of data reported by Ramaswamy et al. (2001). Additionally, GWP as CO_2_e is undefined for the VOC produced in silage. As stated previously, the propensity of each VOC for O_3_ formation in the low-atmosphere (troposphere) has been described by measure of equal benefit incremental reactivity (EBIR), which is used for estimation of GWP based on the radiative forcing equivalent of tropospheric O_3_.

Most reports discussing tropospheric O_3_ do not directly acknowledge a CO_2_e GWP value and instead document a relative change in atmospheric radiative forcing (Iglesias-Suarez et al., 2018). However, a comparison of reported values for increased atmospheric abundance and radiative forcing for CO_2_, CH_4_, and O_3_ (Table 2), amortized for the atmospheric lifetime of O_3_, allows for an estimation of GWP. Ramaswamy et al. (2001) reported atmospheric abundance and radiative forcing for CO_2_ and CH_4_ in the modern era compared with pre-industrial levels. Where the increase in radiative forcing from the respective compounds is normalized to increased atmospheric abundance, CH_4_ is found to contribute to radiative forcing at approximately 27.4 times the magnitude of CO_2_, which agrees with the GWP_100_ value for methane of 25 that is utilized in the United States greenhouse gas inventory (EPA, 2022). Applying the same calculation to estimates of tropospheric abundance and radiative forcing of O_3_, tropospheric O_3_ is found to contribute to radiative forcing at a magnitude of approximately 681.0 times that of CO_2_. For approximate GWP estimation, this relative radiative forcing equivalent for tropospheric O_3_ is amortized to the estimated atmospheric lifetime of the compound, where O_3_ is short-lived with estimated atmospheric lifetime of 22 days (Brasseur et al., 2001; Goldberg et al., 2015). Therefore, estimates for GWP_20_ (20 year horizon) and GWP_100_ (100 year horizon) for tropospheric O_3_ are approximately 2.05 and 0.41 CO_2_e, respectively. These are the constants that are used in Eqs 3 and 4to estimate the GWP of VOC that result in O_3_ formation.

#### 4.1.2. Volatile corrected dry matter loss and respired CO_2_

A complete understanding of the estimates in literature for vcDML (also term D_V_) is further critical for comparing the stoichiometric estimates of CO_2_ production within silage, which are terms C_M_ and C_R_ (Eqs 2 and 8, respectively). Results of model equations demonstrate that vcDML measurements do not agree with stoichiometric projections for CO_2_ production, as evidenced by the mean negative value for model term C_R_. Term C_R_ is fundamentally derived from the difference between empirically measured dry matter loss and estimates of other volatilized mass based on fermentation outcomes. Where respiration occurs to a substantial degree, term C_R_ should be a positive value. Where this term is negative, however, less dry matter loss is observed than should be observed if all projected CO_2_ is emitted from silage, even during the drying process of laboratory analysis. Since term C_R_ is significantly negative, it is hypothesized that the meta-data of Arriola et al. (2021) demonstrate CO_2_ recycling within silage with microbial metabolism within silage facilitating this CO_2_ recycling event.

### 4.2. CO_2_ recycling by silage microorganisms

In the estimation of GWP_20_ and GWP_100_ for silage fermentation, Eq. 2 through Eq. 9 have established three model terms C_M_, C_O_ (as C_O-20_ or C_O-100_), and C_R_. As stated previously, term C_M_ estimates true CO_2_ that is requisitely produced by the decarboxylation of pyruvate to acetyl-CoA during the fermentation of sugars to lactic acid, acetic acid, and minor VOC. This estimation of true carbon dioxide production is compared with an estimate of dry matter loss (or volatile-corrected dry matter loss by substitution of Eq. 6 with a constant) to derive term C_R_, which is an estimate of CO_2_ from respiration. Results in Table 6 document a mean negative value (−1.8 ± 5.5; mean ± SD) for term C_R_, which indicates that less carbon dioxide is produced than the amount estimated by term C_M_.

There is ample evidence in the literature of respiration during the first phase of ensiling, and this paradigm is not disputed here. As reviewed by McDonald (1981), oxygen in an air-tight silo is depleted by respiring plant material within 30 min of ensiling, long before most microorganisms are known to escape the fermentation lag phase. As further reviewed by Ruxton and McDonald (1973), oxygen infiltration to the silo has long been known to promote the growth of aerobic (respiring) microorganisms and to result in higher silage pH, lower lactic acid concentration, temperature rise, and greater dry matter loss (Ruxton and McDonald, 1973). Oxygen-induced respiration in silage has been further modeled to predict dry matter loss (Pitt, 1986), whereas application of oxygen barrier film covering has been proven effective for decreasing dry matter loss in silage in more recent years (Wilkinson and Fenlon, 2014; Wilkinson and Rinne, 2018). Therefore, the authors assert that the negative value for term C_R_ noted above does not describe an absence of respiration, but rather that the amount of CO_2_ estimated to be emitted from silage is less than the amount of CO_2_ that should be derived from the fermentation of sugars to organic acids. This estimation is the mathematical basis for proposed CO_2_ recycling in silage by silage microorganisms.

The above examples further highlight that observations of silage at commercial scale have driven much of the interest in laboratory scale silage research, where differences between these two scales of work are evident. This primary difference is the magnitude of dry matter loss, which is driven by total oxygen exposure. The results of the present work document that at laboratory scale, vcDML was 1.2 ± 3.9% of dry matter. Laboratory scale conditions are typified by small silos constructed of PVC or air-tight plastic material that is tightly sealed and regarded as impermeable to oxygen. No such presumption of absolute impermeability exists at commercial scale, where bunker silos are generally regarded as oxygen-permeable (Pitt, 1986). Reports of commercial-scale dry matter loss are infrequent in the literature, especially compared with laboratory-scale data, but Rota et al. (2012) noted that dry matter loss at commercial scale readily exceeds 7% of dry matter, whereas Pitt (1986) estimated that typical losses can be approximately 1–3% per month of storage. Bolsen et al. (1993) documented dry matter loss in the top 25 cm of silage stored in bunker silos and demonstrated that even polyethylene-covered silage can succumb to dry matter loss approaching 20% of dry matter, which is nearly triple the magnitude of loss for silage at a depth of 75–100 cm from the bunker surface. These data by Bolsen et al. (1993) do support that silage deep in a bunker is much less permeable to gas diffusion than silage near the bunker surface, which applies both to atmospheric oxygen and also to silage gases such as CO_2_, which are produced in the silage.

Although this key difference between laboratory and commercial-scale study systems exists, there is no basis for the fundamental nature of metabolic processes to be different between systems. Certainly, the magnitude of respiration is greater at commercial scale than at laboratory scale, just as has been discussed, but pathways for respiration to CO_2_, or fermentation to organic compounds, abide by the same metabolic course. This fundamental nature of metabolism is captured by Eqs. 2 through 9, which further serve as the basis for linear model Eq. 11 and 12. Therefore, although the metrics of fermentation outcomes and dry matter loss are likely to differ between laboratory and commercial scales, the present model is robust to these differences by means of its basis in the metabolic pathways of fermentation and respiration.

The CO_2_e emissions footprint of silage fermentation, documented in Table 6, was produced from source data entirely from laboratory-scale experiments. Application of the present model for silage fermentation at commercial scale will be dependent on empirical measurements of commercial silage. The authors propose especially that experiments are needed in commercial bunker silos and bag silos to determine vcDML and other model inputs for the sound estimation of GWP_20_ and GWP_100_, as well as for determining the effects of ensiling practices such as packing, covering, use of oxygen barrier film, and application of inoculant.

Just as the physiological basis of fermentation and respiration validate laboratory-scale silage research, term C_R_ in the present modeling effort indicates a mathematical basis for CO_2_ recycling in silage. Such a mechanism is not without precedent. Over 80 years ago, Krebs (1941) reviewed CO_2_ assimilation in heterotrophic bacteria. In the preceding decade, carbonic anhydrase, responsible for catalyzing the reversible hydration of CO_2_ to H_2_CO_3_ (and the further dissociated bicarbonate ion) had been discovered by two independent teams, but not evaluated in bacteria (Meldrum and Roughton, 1933; Stadie and O’Brien, 1933). Much later, carbonic anhydrase was isolated and purified from bacterial species and its properties described (Veitch and Blankenship, 1963; Adler et al., 1972; Brundell et al., 1972). Smith and Ferry (2000) reviewed these and further advancements in prokaryotic carbonic anhydrases, including the discovery of two additional classes (β and γ) of the enzyme that were found to be widespread and dominant in bacteria, compared with the well-described α class of eukaryotic cells (Alber and Ferry, 1994; Smith et al., 1999; Smith and Ferry, 1999). Of note, Smith et al. (1999) demonstrated that although α class carbonic anhydrase enzymes were rare throughout archaeal and bacterial domains, the other classes of the enzyme were widespread, especially in chemolithoautotrophs for which CO_2_ fixation may depend on bicarbonate ion generated from carbonic anhydrase.

Later, Arsène-Ploetze and Bringel (2004) noted that many lactic acid bacteria, including *Lactiplantibacillus* (formerly *Lactobacillus*) *plantarum, Enterococcus faecalis,* and *Enterococcus faecium*, are stimulated by inorganic carbon and are properly described as capnophillic, encoding not only carbonic anhydrase of the prevalent γ class, but also of the rare α class. Each of these organisms are prevalent among native lactic acid bacteria in silages (Langston and Bouma, 1960; Callieri and Malacalza, 1974; Mcdonald, 1981), with *L. plantarum* and *E. faecium* (formerly *Streptococcus faecium*) also used commonly as silage additives.

Yet other researchers have described the mechanisms of CO_2_ transport across microbial cell membranes. Michenkova et al. (2021) recently reviewed several factors that contribute to transmembrane CO_2_ diffusion and permeability. Citing the work of Gutknecht et al. (1977) and Musa-Aziz et al. (2014) alongside new experiments, they demonstrated that intracellular carbonic anhydrase activity could increase the cellular influx of CO_2_. As CO_2_ was converted to carbonic acid within the cell, intracellular CO_2_ decreased. In turn, this increased the CO_2_ gradient between extracellular and intracellular spaces and increased CO_2_ permeability.

Additionally, Michenkova et al. (2021) described cell membrane modifications such as aquaporins that could allow for CO_2_ permeability. Aquaporins are a family of pore proteins that facilitate the selective flux of small molecules across the cell membrane. In Nakhoul et al. (1998) demonstrated that human aquaporin AQP1 acted as a CO_2_ channel. This was the first demonstration of gas movement through a membrane pore. The bacterial glycerol facilitator uptake protein (glpF) is an aquaglyceroroporin, which is an aquaporin subfamily. Protein glpF is known to export lactic acid from the cytosol and is commonly encoded for in lactic acid bacterial genomes. As with other aquaporins, glpF is known not only to transport CO_2_, but is less selective and more permeable to CO_2_ than human AQP1 (Hub and de Groot, 2008). Although these concepts of pure cell physiology have been reviewed extensively in the past (Boron et al., 2011; Endeward et al., 2014; Michenkova et al., 2021), their application to CO_2_ fixation in silage has never been documented.

The results of genome annotation review by these authors mostly agree with Arsène-Ploetze and Bringel (2004), including the absence of carbonic anhydrase in *L. lactis.* However, in the present review, no more than one carbonic anhydrase gene was detected in each of the genomes. Notably, *L. plantarum* was found to have greater carboxylase gene redundancy than the other strains in the analysis.

Higher gene redundancy does not directly translate to higher expression of these genes, but the differences detected in gene presence may indicate the potential for inherent metabolic distinctions between species (Hahn, 2009; Bratlie et al., 2010). Metabolic differences among species of LABs are evident, with *L. plantarum* and *L. lactis* classified as homofermenters of carbohydrates to lactic acid and *L. buchneri* and *L. brevis* classified as heterofermenters of carbohydrates to lactic acid and acetic acid or ethanol (Gänzle, 2015; Wang et al., 2021). Heterofermentation of glucose produces CO_2_ into the silage system, which potentially affects the activity of carbonic anhydrase and the expression of carboxylase genes. However, inoculation of corn silage with heterofermentative bacteria has been shown to result in greater dry matter loss than inoculation with homofermentative LABs, suggesting the released CO_2_ is not entirely recaptured (Tabacco et al., 2011). The observation of carboxylase pathway redundancy in *Lactiplantibacillus plantarum*, a homofermenter, is therefore striking from both aspects of potentially lower CO_2_ production from fermentation (C_M_) and potential CO_2_ recycling (C_R_). Both homo-and heterofementing LAB species have been studied as silage inoculants and these metabolic differences have been extensively covered in other literature (Muck et al., 2018).

Only a small subset of silage-relevant LAB species were analyzed in this study, so whether these pathways are widely distributed among species, either inoculated or native to silage, remains to be determined. As there are currently no transcriptomic studies describing bacterial gene expression during the ensiling process, the prevalence and timing of carbonic anhydrase and carboxylase gene expression also is unknown. Future experiments utilizing quantitative gene expression of carbonic anhydrase and carboxylase enzymes, may help to elucidate the prevalence of this mechanism within the silage environment.

Aside from genomic potential, silage conditions are indeed favorable for CO_2_ recycling pathways. Arsène-Ploetze and Bringel (2004) specifically noted that fermentation environments, especially where association with respiring organisms is prevalent, are rich in inorganic carbon as a substrate for numerous carboxylation reactions in lactic acid bacteria. A possible precedent for a CO_2_ recycling event has been recorded by Li et al. (2017), who documented negative change in silo headspace pressure and respiratory quotient (RQ) < 1 during the first 5 h of ensiling, albeit net positive change in CO_2_ concentration in the silo. Li et al. (2017) attributed these results to the dissolution of CO_2_ in silage water, whereby the greater solubility of CO_2_ compared with O_2_ was purported to account for the loss of gaseous mass from the headspace.

In essence, this observation identified that less CO_2_ was produced in the headspace than what should have been produced if RQ = 1. The outcomes of the present modeling effort also support this notion, but the primary result of the present findings is that model term C_R_ is consistently negative, and this outcome is tied to empirical findings of dry matter loss, which should account for dissolved CO_2_ in silage water by virtue of the drying process. It is important to note that Li et al. (2017) proposed CO_2_ dissolution kinetics by means of carbonic acid formation and further dissociation to bicarbonate ion, but there is ample precedent for simple diffusion or channel-mediated transfer of gaseous CO_2_ in the biological systems, as described above. The purpose of discussing these diffusion kinetics is to further extend that gaseous CO_2_, if dissolved into silage water, should be accounted for in our estimation of term C_R_. Therefore, we extend that this discrepancy is not solely accounted for by dissolution kinetics, but rather that it is highly likely for CO_2_ to be recycled, especially by means of intracellular carbonic anhydrase and carboxylase activities.

In addition to farm-scale work and transcriptomic studies already proposed, future work must aim to quantify the proportion of available CO_2_ in a silage environment that is recycled by such pathways in silage microorganisms. A likely means for such a study is the use of isotopically-labelled CO_2_ or substrates in a silage experiment. Theoretically, the value of term C_R_ can be justified by measuring the proportion of CO_2_ recycled by cells as a proportion of respired CO_2_ if both respiration and CO_2_ assimilation can be accounted for. Experiments utilizing isotopic carbon are likely to be fruitful in this regard.

For silage microorganisms with membranes permeable to CO_2_, the influx of CO_2_ to the cytosol pushes equilibrium to favor the formation of carbonic acid and the further dissociated bicarbonate ion. As a charged ion, bicarbonate is less permeable to the cell membrane than dissolved CO_2_. Equilibrium favoring dissociated bicarbonate ion is especially favored in the near-neutral cytosol as the p*K*_a_ of carbonic acid has been empirically determined at 3.49 ± 0.05 (Pines et al., 2016), a value revised from previous calculations (Tossell, 2005). In fermenting microorganisms, the CO_2_-rich environment of silage similarly favors CO_2_ diffusion across the cell membrane to the cytosol, where equilibrium again favors formation of carbonic acid and bicarbonate ion, especially in microorganisms utilizing carboxylase pathways. This proposed mechanism fully agrees with the high prevalence of capnophillic lactic acid bacteria that are commonly observed in silages. Therefore, where GWP estimates of silage fermentation are considered, the documentation of negative values in the statistical uncertainties does not disagree with precedent in the literature for CO_2_ recycling by silage microorganisms. Manipulation of silage bacteria and the use of optimal ensiling procedures holds great promise for decreasing silage CO_2_e emissions and perhaps even the sequestration of inorganic carbon.

### 4.3. Implications of silage for national emissions inventories

It was previously noted that silage fermentation is excluded from national emissions inventories that are reported to the UNFCC. The USDA reported 130.3 Mt. of corn silage harvested from United States cropland in 2021 (NASS, USDA, 2022). Arriola et al. (2021) reported mean dry matter of 32.0% (data not shown), for an estimated 41.6 Mt. of corn silage dry matter. With GWP_20_ of 1.9 ± 5.6% of silage dry matter calculated in the present work, corn silage fermentation is projected to account for 791 kt of CO_2_e on a 20 year horizon. On a 100 year horizon (GWP_100_ of 0.2 ± 5.5%), CO_2_e emissions total approximately 83 kt, although the 95% confidence interval demonstrates that this value is not significantly different from 0. For additional perspective, the CO_2_e footprint of corn silage production has been estimated at approximately 200 g CO_2_e per kg dry matter, or approximately 20% by mass (Adom et al., 2012).

The low 95% confidence limit extends the possibility of GWP_100_ from corn silage fermentation to -10.8% of silage dry matter. In practice, this value does not seem sensible or achievable, considering that respired losses from silage have long been considered inevitable. However, as discussed, silage studied at the laboratory scale clearly demonstrates the possibility for negative vcDML and inorganic carbon recycling. Rather than quantify the value of U.S. corn silage as a potential emissions sink at the extreme low value of the 95% confidence interval, it shall suffice to simply project the value of incrementally improved silage preservation. In Eq. 12 above, a decrease in vcDML of 1 percentage point (i.e., from 2 to 1% vcDML) equates to a 1 percentage point decrease in GWP_100_ if other fermentation metrics such as organic acids and ethanol remain constant. This magnitude of change is within the bounds of current silage preservation techniques such as packing, covering, and inoculating silage (Goeser et al., 2015; Borreani et al., 2018). At the scale of U.S. corn silage production, one such increment would amount to a reduction of 416 kt CO_2_e.

In the 2020 U.S. national emissions inventory (EPA, 2022), the agricultural sector accounted for 594.7 Mt. of CO_2_e emissions. The estimate for GWP_100_ from corn silage fermentation at national scale (83 kt and not statistically different from zero) is far below any other itemized source and is therefore not a meaningful CO_2_e source. Optimal silage preservation practices that sink inorganic carbon in silage could be meaningful to the national inventory if a net reduction in CO_2_e emissions of approximately 1 to 2 Mt., which is the approximate threshold for itemization, can be achieved.

### 4.4. Conclusion

The key conclusions drawn from this work encompass logical consideration of silage fermentation emissions, mean and variance estimates for silage fermentation outcomes, estimates and uncertainties for CO_2_e emissions from silage, a linear equation for estimating emissions from singular silage samples, and an established precedent for CO_2_ recycling by silage microorganisms. CO_2_e emissions from silage are the sum of CO_2_ produced as the metabolic outcome of pyruvate decarboxylation, O_3_ resulting from the evaporation of VOC, and respiration. The review by these authors of previously published meta-analyses indicates that mean and variance (SD) estimates for silage fermentation outcomes (% of dry matter) are lactic acid concentration of 5.4 ± 2.2%, acetic acid concentration of 1.6 ± 0.8%, ammonia concentration of 0.2 ± 0.3%, ethanol concentration of 1.0 ± 0.8%, and volatile corrected dry matter loss of 1.1 ± 3.8%. Estimates for CO_2_e emissions produced from mock data were (for GWP_20_) 1.9 ± 5.6% (mean ± SD) with 95% confidence limits of-9.2 to 13.1% of dry matter. Similarly, GWP_100_ was estimated at 0.2 ± 5.5% of dry matter with 95% confidence limits of-10.8 to 11.2% of dry matter. The linear equations for estimating CO_2_e emissions from silage, where all inputs and outputs are expressed in mg kg^−1^, were (for GWP_20_; Eq. 11):


(11b) $$\begin{array}{l} {\mathrm{GWP}}_{20}=-3626.1-0.04343A+0.8011\;-\\ \;\;\;\;\;\;\;\;\;\;\;\;\;\;\;\;\;\;\;\;\;\;\;0.03173L+1.46573D_{V} \end{array}$$

and for GWP_100_; Eq. 12:


(12b) $$\begin{array}{l} {\mathrm{GWP}}_{100}=-8526.1-0.22403A-0.11963E-\\ \;\;\;\;\;\;\;\;\;\;\;\;\;\;\;\;\;\;\;\;\;\;\;\;0.03173L+1.46573D_{V} \end{array}$$

where A is acetic acid, E is ethanol, L is lactic acid, and D is volatile corrected dry matter loss. Finally, where silage microorganisms are capable of recycling CO_2_, fermented silage as animal feed has significant potential for decreased emissions, even so far as a carbon sink, by the application and manipulation of silage bacteria.

## Data availability statement

Processed data in this study are deposited in the GitHub repository and are available to the public at github.com/lucasakrueger/doi.10.3389.fmicb.2022.1092315.

## Author contributions

LKr conceptualized the study and obtained data pertaining to emissions modelling, performed all calculations and statistics pertaining to the emissions model, and wrote the first draft of the manuscript. LKo obtained and mined genomic information from silage inoculant bacterial strains. LKr, LKo, DJ, and DS were involved in writing and editing successive drafts of the manuscript. All authors contributed to the article and approved the submitted version.

## Funding

The work was funded by Agri-King, Inc. The funder had the following involvement with the study: study design, data analysis, decision to publish, and preparation of the manuscript.

## Conflict of interest

LKr, LKo, DJ, and DS were employed by Agri-King, Inc., which markets and sells a silage additive under the brand name of Silo-King^®^.

## Publisher’s note

All claims expressed in this article are solely those of the authors and do not necessarily represent those of their affiliated organizations, or those of the publisher, the editors and the reviewers. Any product that may be evaluated in this article, or claim that may be made by its manufacturer, is not guaranteed or endorsed by the publisher.

## Footnotes

^1^ http://www.wessa.net/rwasp_boxcoxnorm.wasp

^2^ https://www.socscistatistics.com/utilities/normaldistribution/default.aspx
