# Corrigendum: Carbon dioxide equivalent emissions from corn silage fermentation

**DOI:** 10.3389/fmicb.2023.1176518

**Published:** 2023-03-14

**Authors:** Lucas A. Krueger, Lucas R. Koester, David F. Jones, David A. Spangler

**Affiliations:** Department of Research, Development, and Biotechnology, Agri-King, Inc., Fulton, IL, United States

**Keywords:** silage, greenhouse gases, fermentation, carbon dioxide equivalent emissions, sustainability, carbonic anhydrase, lactic acid bacteria

In the published article, there was an error.

A typographical error was made to Equation (2) that is presented in the paper. This error was typographical only and does not affect the underlying calculations.

A correction has been made to **2. Materials and methods**, *2.2.1 Direct CO*_2_ *emissions from fermentation*, Equation (2). This sentence previously stated:


$$\begin{array}{l} C_{M}=\;0.65\;\times \;\left[\left(\text{A}\frac{M_{A}}{M_{C}}\right)+\;\left(\text{E}\frac{M_{E}}{M_{C}}\right)+\;\left(1.6\;\times \;10^{3}\right)\right] \end{array}$$

The corrected sentence appears below:


$$\begin{array}{l} C_{M}=\;0.65\;\times \;\left[\left(\text{A}\frac{M_{C}}{M_{A}}\right)+\;\left(\text{E}\frac{M_{C}}{M_{E}}\right)+\;\left(1.6\;\times \;10^{3}\right)\right] \end{array}$$

The authors apologize for this error and state that this does not change the scientific conclusions of the article in any way. The original article has been updated.

