# Supplementary material for: Carbon dioxide equivalent emissions from corn silage fermentation
Source: Front Microbiol. 2023 Jan 9;13:1092315. doi: 10.3389/fmicb.2022.1092315 (PMC9869070; doi:10.3389/fmicb.2022.1092315)
Supplement: Supplementary file 1 [file Table_1.DOCX]

Supplementary tables:

**Table S1:** NCBI GenBank reference sequence for freely available genomes.

| **Genome** | **Reference Sequence** |
| --- | --- |
| L.plantarum (SK151) | NZ_CP030105.1 |
| L.lactis (LAC640) | NZ_CP059048.1 |
| L.brevis (NPS_QW_145) | NZ_CP015398.1 |
| L.buchneri (ATCC4005) | GCF_018314255.1 |

**Table S2:** NCBI BLASTp results for ORF sequences of query matches.

| **Organism** | **ORF** | **NCBI description** | **Query coverage** | **Percent identity** | **AA length** | **Accession** |
| --- | --- | --- | --- | --- | --- | --- |
| L.plantarum (SK151) | >CJIJOCHA_00620 D/L-lactic acid transporter (glpF) | aquaporin family protein [Lactiplantibacillus plantarum] | 100% | 100.00% | 242 | WP_063846036.1 |
| L.plantarum (SK151) | >CJIJOCHA_02535 Glycerol facilitator-aquaporin gla (glpF) | aquaporin family protein [Lactiplantibacillus plantarum] | 100% | 100.00% | 255 | WP_157262796.1 |
| L.plantarum (SK151) | >CJIJOCHA_02889 Glycerol uptake facilitator protein (glpF) | aquaporin family protein [Lactobacillaceae] | 100% | 100.00% | 235 | WP_003641766.1 |
| L.plantarum (SK151) | >CJIJOCHA_03068 Glycerol uptake facilitator protein (glpF) | aquaporin family protein [Lactiplantibacillus plantarum] | 100% | 99.58% | 240 | WP_114692872.1 |
| L.plantarum (SK151) | >CJIJOCHA_02835 D/L-lactic acid transporter (glpF) | aquaporin family protein [Lactobacillaceae] | 100% | 100.00% | 238 | WP_003643656.1 |
| L.plantarum (SK151) | >CJIJOCHA_01966 Carbonic anhydrase (cah) | carbonic anhydrase family protein [Lactiplantibacillus] | 100% | 100.00% | 211 | WP_003642580.1 |
| L.plantarum (SK151) | >CJIJOCHA_01939 Carbamoyl-phosphate synthase small chain (carA) | carbamoyl phosphate synthase small subunit [Lactiplantibacillus plantarum] | 100% | 99.45% | 364 | WP_161324529.1 |
| L.plantarum (SK151) | >CJIJOCHA_00110 Carbamoyl-phosphate synthase arginine-specific small chain (carA) | carbamoyl phosphate synthase small subunit [Lactiplantibacillus plantarum] | 100% | 99.44% | 355 | WP_047672727.1 |
| L.plantarum (SK151) | >CJIJOCHA_01234 Carbamoyl-phosphate synthase small chain (carA) | carbamoyl phosphate synthase small subunit [Lactiplantibacillus] | 100% | 100.00% | 360 | WP_003640511.1 |
| L.plantarum (SK151) | >CJIJOCHA_01938 Carbamoyl-phosphate synthase large chain (carB) | carbamoyl-phosphate synthase large subunit [Lactiplantibacillus] | 100% | 100.00% | 1058 | WP_013355826.1 |
| L.plantarum (SK151) | >CJIJOCHA_00109 Carbamoyl-phosphate synthase arginine-specific large chain (carB) | carbamoyl-phosphate synthase large subunit [Lactiplantibacillus plantarum] | 100% | 100.00% | 1020 | WP_112297224.1 |
| L.plantarum (SK151) | >CJIJOCHA_01235 Carbamoyl-phosphate synthase large chain (carB) | ATP-grasp domain-containing protein [Lactiplantibacillus plantarum] | 100% | 100.00% | 853 | WP_022637989.1 |
| L.plantarum (SK151) | >CJIJOCHA_01518 Pyruvate carboxylase (pyc) | pyruvate carboxylase [Lactiplantibacillus] | 100% | 100.00% | 1143 | WP_011101664.1 |
| L.plantarum (SK151) | >CJIJOCHA_01082 Acetyl-coenzyme A carboxylase carboxyl transferase subunit alpha (accA) | acetyl-CoA carboxylase carboxyl transferase subunit alpha [Lactiplantibacillus plantarum] | 100% | 100.00% | 256 | WP_112297271.1 |
| L.plantarum (SK151) | >CJIJOCHA_00183 Acetyl-coenzyme A carboxylase carboxyl transferase subunit alpha (accA1) | acetyl-CoA carboxylase, carboxyl transferase subunit alpha [Lactiplantibacillus plantarum subsp. plantarum ATCC 14917 = JCM 1149 = CGMCC 1.2437] | 100% | 100.00% | 264 | KRL33884.1 |
| L.plantarum (SK151) | >CJIJOCHA_01078 Biotin carboxyl carrier protein of acetyl-CoA carboxylase (accB) | acetyl-CoA carboxylase biotin carboxyl carrier protein subunit [Lactiplantibacillus] | 100% | 100.00% | 153 | WP_003640414.1 |
| L.plantarum (SK151) | >CJIJOCHA_00180 hypothetical protein (accB1) | acetyl-CoA carboxylase biotin carboxyl carrier protein subunit [Lactiplantibacillus plantarum] | 100% | 99.25% | 133 | WP_161324205.1 |
| L.plantarum (SK151) | >CJIJOCHA_03059 Biotin carboxyl carrier protein of acetyl-CoA carboxylase (accB3) | acetyl-CoA carboxylase biotin carboxyl carrier protein subunit [Lactiplantibacillus] | 100% | 100.00% | 190 | WP_013355176.1 |
| L.plantarum (SK151) | >CJIJOCHA_01080 Biotin carboxylase (accC) | acetyl-CoA carboxylase biotin carboxylase subunit [Lactiplantibacillus] | 100% | 100.00% | 462 | WP_003640416.1 |
| L.plantarum (SK151) | >CJIJOCHA_00181 Biotin carboxylase (accC1) | acetyl-CoA carboxylase biotin carboxylase subunit [Lactiplantibacillus] | 100% | 100.00% | 440 | WP_013355226.1 |
| L.plantarum (SK151) | >CJIJOCHA_01081 Acetyl-coenzyme A carboxylase carboxyl transferase subunit beta (accD) | acetyl-CoA carboxylase, carboxyl transferase subunit beta [Lactiplantibacillus plantarum ST-III] | 100% | 100.00% | 281 | ADN98555.1 |
| L.plantarum (SK151) | >CJIJOCHA_00182 Acetyl-coenzyme A carboxylase carboxyl transferase subunit beta (accD1) | acetyl-CoA carboxylase carboxyltransferase subunit beta [Lactiplantibacillus] | 100% | 100.00% | 267 | WP_003640918.1 |
| L.plantarum (SK151) | >CJIJOCHA_00179 3-oxoacyl-[acyl-carrier-protein] synthase 3 (fabH) | ketoacyl-ACP synthase III [Lactiplantibacillus plantarum] | 100% | 99.69% | 323 | WP_072535677.1 |
| L.plantarum (SK151) | >CJIJOCHA_01073 3-oxoacyl-[acyl-carrier-protein] synthase 3 (fabH) | ketoacyl-ACP synthase III [Lactiplantibacillus] | 100% | 99.70% | 328 | WP_011101499.1 |
| L.lactis (LAC640) | >NNLFFCON_01266 Glycerol uptake facilitator protein (glpF) | aquaporin family protein [Lactococcus lactis] | 100% | 100.00% | 245 | WP_129881659.1 |
| L.lactis (LAC640) | >NNLFFCON_00217 Glycerol uptake facilitator protein (glpF) | aquaporin family protein [Lactococcus lactis] | 100% | 100.00% | 238 | WP_129881313.1 |
| L.lactis (LAC640) | >NNLFFCON_01608 Carbamoyl-phosphate synthase small chain (carA) | carbamoyl phosphate synthase small subunit [Lactococcus lactis] | 100% | 100.00% | 357 | WP_058204855.1 |
| L.lactis (LAC640) | >NNLFFCON_01393 Carbamoyl-phosphate synthase large chain (carB) | carbamoyl-phosphate synthase large subunit [Lactococcus lactis] | 100% | 100.00% | 1064 | WP_168784501.1 |
| L.lactis (LAC640) | >NNLFFCON_00581 Pyruvate carboxylase (pyc) | pyruvate carboxylase [Lactococcus lactis] | 100% | 100.00% | 1137 | WP_226319375.1 |
| L.lactis (LAC640) | >NNLFFCON_00742 Acetyl-coenzyme A carboxylase carboxyl transferase subunit alpha (accA) | acetyl-CoA carboxylase carboxyl transferase subunit alpha [Lactococcus lactis] | 100% | 99.62% | 264 | WP_021722951.1 |
| L.lactis (LAC640) | >NNLFFCON_00738 Biotin carboxyl carrier protein of acetyl-CoA carboxylase (accB) | acetyl-CoA carboxylase biotin carboxyl carrier protein [Lactococcus] | 100% | 98.71% | 155 | WP_139916356.1 |
| L.lactis (LAC640) | >NNLFFCON_00740 Biotin carboxylase (accC) | acetyl-CoA carboxylase biotin carboxylase subunit [Lactococcus lactis] | 100% | 100.00% | 455 | WP_012897458.1 |
| L.lactis (LAC640) | >NNLFFCON_00741 Acetyl-coenzyme A carboxylase carboxyl transferase subunit beta (accD) | acetyl-CoA carboxylase, carboxyltransferase subunit beta [Lactococcus] | 100% | 100.00% | 288 | WP_003132516.1 |
| L.lactis (LAC640) | >NNLFFCON_00733 3-oxoacyl-[acyl-carrier-protein] synthase 3 (fabH) | ketoacyl-ACP synthase III [Lactococcus lactis] | 100% | 100.00% | 325 | WP_129881511.1 |
| L.brevis (NPS_QW_145) | >HEBPGAIP_02242 Glycerol facilitator-aquaporin gla (glpF) | aquaporin family protein [Limosilactobacillus fermentum] | 100% | 99.55% | 240 | WP_003681159.1 |
| L.brevis (NPS_QW_145) | >HEBPGAIP_01972 Glycerol uptake facilitator protein (glpF) | aquaporin family protein [Levilactobacillus brevis] | 100% | 100.00% | 235 | WP_011668322.1 |
| L.brevis (NPS_QW_145) | >HEBPGAIP_00180 Carbonic anhydrase (cah) | carbonic anhydrase family protein [Levilactobacillus brevis] | 100% | 100.00% | 212 | WP_024526096.1 |
| L.brevis (NPS_QW_145) | >HEBPGAIP_01462 Carbamoyl-phosphate synthase small chain (carA) | carbamoyl phosphate synthase small subunit [Levilactobacillus brevis] | 100% | 100.00% | 358 | WP_021742568.1 |
| L.brevis (NPS_QW_145) | >HEBPGAIP_01365 Acetyl-coenzyme A carboxylase carboxyl transferase subunit alpha (accA) | acetyl-CoA carboxylase carboxyl transferase subunit alpha [Levilactobacillus brevis] | 100% | 100.00% | 255 | WP_065201353.1 |
| L.brevis (NPS_QW_145) | >HEBPGAIP_01361 Biotin carboxyl carrier protein of acetyl-CoA carboxylase (accB) | acetyl-CoA carboxylase biotin carboxyl carrier protein subunit [Levilactobacillus brevis] | 100% | 100.00% | 141 | WP_021742581.1 |
| L.brevis (NPS_QW_145) | >HEBPGAIP_01363 2-oxoglutarate carboxylase small subunit (accC) | acetyl-CoA carboxylase biotin carboxylase subunit [Levilactobacillus brevis] | 100% | 100.00% | 473 | ARN92471.1 |
| L.brevis (NPS_QW_145) | >HEBPGAIP_01364 Acetyl-coenzyme A carboxylase carboxyl transferase subunit beta (accD) | acetyl-CoA carboxylase carboxyltransferase subunit beta [Levilactobacillus brevis] | 100% | 100.00% | 277 | WP_024525316.1 |
| L.brevis (NPS_QW_145) | >HEBPGAIP_01356 3-oxoacyl-[acyl-carrier-protein] synthase 3 (fabH) | ketoacyl-ACP synthase III [Levilactobacillus brevis] | 100% | 100.00% | 327 | WP_011667660.1 |
| L.buchneri (ATCC4005) | >PCFFGLNM_01866 Glycerol uptake facilitator protein (glpF) | aquaporin family protein [Lentilactobacillus buchneri] | 100% | 100.00% | 234 | WP_013727474.1 |
| L.buchneri (ATCC4005) | >PCFFGLNM_01780 Carbonic anhydrase (cah) | carbonic anhydrase family protein [Lentilactobacillus buchneri] | 100% | 100.00% | 210 | WP_013727391.1 |
| L.buchneri (ATCC4005) | >PCFFGLNM_00966 Carbamoyl-phosphate synthase arginine-specific small chain (carA) | carbamoyl phosphate synthase small subunit [Lentilactobacillus buchneri] | 100% | 100.00% | 352 | WP_211772871.1 |
| L.buchneri (ATCC4005) | >PCFFGLNM_02353 Carbamoyl-phosphate synthase small chain (carA) | carbamoyl phosphate synthase small subunit [Lentilactobacillus buchneri] | 100% | 100.00% | 363 | WP_013727897.1 |
| L.buchneri (ATCC4005) | >PCFFGLNM_00967 Carbamoyl-phosphate synthase arginine-specific large chain (carB) | carbamoyl-phosphate synthase large subunit [Lentilactobacillus buchneri] | 100% | 100.00% | 1021 | WP_056939184.1 |
| L.buchneri (ATCC4005) | >PCFFGLNM_00486 Acetyl-coenzyme A carboxylase carboxyl transferase subunit alpha (accA) | acetyl-CoA carboxylase carboxyl transferase subunit alpha [Lentilactobacillus buchneri] | 100% | 100.00% | 256 | WP_056939203.1 |
| L.buchneri (ATCC4005) | >PCFFGLNM_00490 Biotin carboxyl carrier protein of acetyl-CoA carboxylase (accB) | acetyl-CoA carboxylase biotin carboxyl carrier protein [Lentilactobacillus buchneri] | 100% | 100.00% | 157 | WP_014940304.1 |
| L.buchneri (ATCC4005) | >PCFFGLNM_00488 Biotin carboxylase (accC) | acetyl-CoA carboxylase biotin carboxylase subunit [Lentilactobacillus buchneri] | 100% | 100.00% | 459 | WP_013728442.1 |
| L.buchneri (ATCC4005) | >PCFFGLNM_00487 Acetyl-coenzyme A carboxylase carboxyl transferase subunit beta (accD) | acetyl-coenzyme A carboxylase carboxyl transferase subunit beta [Lentilactobacillus buchneri DSM 20057] | 97% | 100.00% | 274 | KRK67173.1 |
| L.buchneri (ATCC4005) | >PCFFGLNM_00495 3-oxoacyl-[acyl-carrier-protein] synthase 3 (fabH) | ketoacyl-ACP synthase III [Lentilactobacillus buchneri] | 100% | 100.00% | 327 | WP_013728449.1 |

**Table S3:** ORF sequences of query matches from all genomes.

| Organism | ORF |
| --- | --- |
| L.plantarum (SK151) | >CJIJOCHA_00620 D/L-lactic acid transporter (glpF) |
| L.plantarum (SK151) | MIHQLLAEFMGTALMIIFGVGVHCSEVLKGTKYRGSGHIFAITTWGFGITIALFIFGNVCINPAMVLAQCILGNLSWSLFIPYSVAEVLGGVVGAVIVWIMYADHFAASADEISPITIRNLFSTAPAVRNLPRNFFVEFFDTFIFISGILAISEVKTPGIVPIGVGLLVWAIGMGLGGPTGFAMNLARDMGPRIAHAILPIKNKADSDWQYGIIVPGIAPFVGAACAALFMHGFFGIG |
| L.plantarum (SK151) | >CJIJOCHA_02535 Glycerol facilitator-aquaporin gla (glpF) |
| L.plantarum (SK151) | MTGSWEARYAAEFFGTLILVLLGNGAVANAFLKNTTGNDDPGLANGGWLLVASGYGLGVMLPAMMFGSISGNHLNPAITIGQAVIGIFPWAHVAPYLIWQFLGAIAGQCLILALYWPHYRQTTDNEAVLGTFATSDHANSQLNGFVTEMVGTAVLIFGAMGLYRGMFFHQNIDIANIGVGLLIAAMVISLGGPTGPALNPARDLGPRLVHALLPVPNKGSSHWEYSWVPVVAPIVGAVIGIWIYKIFFGL |
| L.plantarum (SK151) | >CJIJOCHA_02889 Glycerol uptake facilitator protein (glpF) |
| L.plantarum (SK151) | MHGFLGEFLGTMVLIVFGVGSGAAMNLKGNYARHQNWTFICLAWGLAVTFGVYVAGQFGSDGHLNPAVTVGFALFGYLPMANVWPYLLGQFLGAFIGAVIVIIQYYPHFQAAKTAADGNQVGIFATGPAISNPVFNFLSETIATFFFIFVLLNLGNFTQGLKPLMVGLLIVVVGQTLGGTTGFAINPARDWAPRLAYTILPVPNKGLANWGYAWVPMFGPLLGGILAAGLETIIS |
| L.plantarum (SK151) | >CJIJOCHA_03068 Glycerol uptake facilitator protein (glpF) |
| L.plantarum (SK151) | MMKDPLALQLLGEFLGTFILILLGDGVVAGVTLNKSKAQNAGWVAITLGWGFAVTMGVYASSFMSPAHLNPAVSLGMAVAGKFPWAYVIPYSAAQIAGGVIGGLVVWLHYYPHWQATKDAGAILGIFATGPGIRRYFWNFISEVIGTFVLVFGLLAFTKGQFTAGLNPIVVGILIIAIGLSLGGTTGYAINPARDLGPRIAHAVLPIANKGTSDWAYSWVPIAGPLVGGALGALLFNVLP |
| L.plantarum (SK151) | >CJIJOCHA_02835 D/L-lactic acid transporter (glpF) |
| L.plantarum (SK151) | MVHQLIAEFMGTALMIIFGVGVHCSSVLKGTKYRGSGHIFAITTWGFGISVALFIFGNVCINPAMVLAQCLLGNIAWSLFIPYSVAEVLGGVVGSVIVWIMYADHFKASTDEISPITIRNLFCTAPAVRNLPRNFFVELFDTFIFISGILAISEIKTPGIVPIGVGLLVWAIGMGLGGPTGFAMNLARDMGPRIAHAILPIANKADSDWQYGIIVPGIAPFVGAAIAAWFMHGFFGIN |
| L.plantarum (SK151) | >CJIJOCHA_01966 Carbonic anhydrase (cah) |
| L.plantarum (SK151) | MTFDYAHQTDWPMTSGQHQSPIDIQTQHVQSSQLSAINWRGLYQAKTITGEATTLKASGIGAAYLNDRDFAFQQIHFHTPAEHLIDGQAAPIEWHLVHQSATGQLAVVAVFGRIGKPNSSLQGLLDQFTAQTSHELTTPVNLTELLPDTGTVYHYLGSLTTPPLSETVEWYVCADTVMIGEQQLAAYQQLFAANNRKIQPLNDRPIIAERF |
| L.plantarum (SK151) | >CJIJOCHA_01939 Carbamoyl-phosphate synthase small chain (carA) |
| L.plantarum (SK151) | MKRYLVLEDGTIYPGTGFGATTATVGELVFNTGMSGYQESITDQSYNGEILMFTYPLIGNYGINRDDHESIKPTCKGVVVHEVARRASNWRNAQSLDDYLKQNAIPGIMDIDTRAVTKHIRTKGAMKATIVDNVLPDTVDRLKITELNRAVVAQSSTNNAYPNPATGPNVVVVDFGLKHSILRELAKRQCNLTVLPYNTTASEIMALNPDGVMLTNGPGDPKDVPGALEMIREVEKHVPLFGICLGHQLFALANGADTFKMKFGHRGFNHPVREIATGRIDFTSQNHGYAVDRDSLAQTDLLITHEEINDGTVEGLRHRDYAAFSVQYHPDAAPGPHDADHIFDEFIDLMAANQATQKGSQFNA |
| L.plantarum (SK151) | >CJIJOCHA_00110 Carbamoyl-phosphate synthase arginine-specific small chain (carA) |
| L.plantarum (SK151) | MNKYLTLADGTQWIGTAIGDCQLEAAGRIVFNTGMTGYQETLTDPSYLNQMIAFTYPLIGNYGIDPTVAQAPTIGAQAIIVHELATFNDHYTSRQSLASFLTIHHVAGIEGVDTRDLTIHIRQTGAQMAILSNHPITDFEAQLATFAPQVLTATPLPVATTTIRPRVAILNFGEKAAITAELQARGADVVVLPPTASLKAVAAYHPDGILLSNGPGDPTDYHTYLATIRQLAQRYPLAGICLGHQLIALAYGARTYQLSFGHHGLNHPVQACADGRIIMTSQNHDYAVDPASIKGTPLIVTHTELNDGSIEGLRLPHQAVMSVQFHPEAHPGPQEAGQFFDDFLLTIQKEAVVNA |
| L.plantarum (SK151) | >CJIJOCHA_01234 Carbamoyl-phosphate synthase small chain (carA) |
| L.plantarum (SK151) | MADRYLILEDGSAYLGEGFGSPAVSTGEIVVNTSMTGYQEIITNQIYHNQIVAFTQPTIGSYGINHDSYESILPTVKGVVVRDVASISTNRQRRWSLDQYLKQQNIPGISHIDTRHLAKQLRASGPMKASIVDVADAHAFDQLGATVLTNQQVAAVATPKPFPNPGTGLNVVVIDFGLKHGILRELSKRACNVTVLPYTATTEEILNLDPDGVILSTGPGKPQDLPASVTEMIKNIQNRVPLFAIGLGHELFAMANGAQIMKLSPEHHGANHPIREVITNQIIYAAQGQGFAVDADTVDRNKLITTFVDLIDGTIQGLRLRDFPAFSVQFFPDGAPGPTETRDIFDEFVESMQQARGPIW |
| L.plantarum (SK151) | >CJIJOCHA_01938 Carbamoyl-phosphate synthase large chain (carB) |
| L.plantarum (SK151) | MPKRTDIHKIMVIGSGPIIIGQAAEFDYSGTQACLALKELDYEVVLVNSNPATIMTDKEIADQVYLEPITLEFVSQILRKEHPDAILPTLGGQQGLNMAMELSKSGILDELHIELLGTKLSAIDQAEDREQFKALMEELGEPVPASGIARTVDEALAFAEQAGYPVIVRPAFTMGGTGGGIAETPQQLHDITENGLALSPVTQVLIEQSIAGYKEIEFEVMRDAADNAMVVCNMENFDPVGIHTGDSIVYAPVQTLADREVQLLRDAALKIIRALKIEGGCNVQLALDPNSFNYYIIEVNPRVSRSSALASKATGYPIAKMAAKIAVGLHLDEIKNPVTGTTYAEFEPALDYVVCKIPRWPFDKFTHADRRLGTQMKATGEVMAIGRNIEEATLKAVRSLEIGVHHVEEPTLRSVDDDVLSDKLIHAQDDRLFYLTEAIRRGYPIDELAELTKINVFFLDKLLHIIEIEQALRTHTDDIETLTVAKRNGFADQTVADYWHETIDQVRDFRLAHKLAPVYKMVDTCAGEFASETPYYYGTYEFENESIVTKRPSVLVLGSGPIRIGQGVEFDYATVHSVKAIQKAGYEAIIMNSNPETVSTDFSVSDKLYFEPLTIEDVLNVIELEKPVGVIVQFGGQTAINLAKPLADHGIKILGTSVADVNRAEDRDEFDKVIKALAIPQPAGDTASDEATALAIADKLGYPVLVRPSYVLGGRAMEIVKKRTDLDYYMHNAVKVSHDHPVLVDSYLVGKECEVDAICDGQTVLIPGIMEHIERAGVHSGDSMAVYPPQSLSAAVQAQIVDYTEKLAIALNCVGMMNIQFVIHDDQVYVIEVNPRASRTVPFLSKVTNIPMAQVATRAILGQSLAEQGYQTGLVTPGPLVHVKAPVFSFSKLNRVDSLLGPEMKSTGEVMGSDVTMAKALYKAFEAAKLHVPSHGNVLLTVRDEDKPETVALAKRFHALGYQLLATRGTATALTTHGLPVTTVDKIDSGEHDLLHRMEAGEVQVVINTVSDEEQAENDGTLIRNTSIMHGIPLFTALDTVAAILQVRESQSFVTQAL |
| L.plantarum (SK151) | >CJIJOCHA_00109 Carbamoyl-phosphate synthase arginine-specific large chain (carB) |
| L.plantarum (SK151) | MPKNNAIHSIAVIGSGPIKIGQAAEFDYAGTQACLSLKAAGYHVILINSNPATIMTDTATADEVYLEPLTLTSVTKILRAAHPDALLPTLGGQTGLNLAMALDQAGVLNELKIQLLGTSLATINQAEDRAAFKTLMKRLHQPIPASTTVHHVTSALSFAEKIGYPVIVRPAFTLGGSGGGIANNAAELTQTLQRGLTMSPVTECLIEQSIAGFKEIEFEVMRDNQGTKIIVCSMENFDPVGIHTGDSIVYAPVQTLTDTEYQQLRTAALTIVEALDIRGGCNVQLAQDPNSRQYYVIEVNPRVSRSSALASKATGYPIAKIAADIAIGLNLSEIKNPVTQTTYAAFEPALDYVVAKIPRFAFDKFPTADAHLGTQMKAMGEVMAIGSTLEEATLKAIASLEIDPKTQASLTPDHHVTTTEYIDQLTHPTDQRLFYLLAALQAGWPLAKLATLTQITPFFLSKLQHIAQLIRNIKQVPTSQHLLSAKKYGVSLATMAHYAQTSVATIAAMTADLPFVYKMVDTCAGEFASVTPYFYSTAFGQTNESHPLGHSILVLGSGPIRIGQGIEFDYTTVHCVKAIQQAGYHAIIVNNNPETVSTDFSTSDKLYFEPLTIERLMPIIALEQPAGVIVQFGGQTAINLAYQLTKLGVTVLGTSVSATDLTEDRQSFADCLRLLKIAQAAGTTVTELSGARQAAHAIGYPLLVRPSFVLGGRAMAIVHNDHELTPVIKSAVAAGHGAPILMDQYLAGTECEVDVLSDGTSCFIPGIMEHIEGAGVHSGDSITVYPPQHLSPAVQDKIVTIATKLAQHLHCVGMMNIQFVVTDDVYVIDVNPRASRTVPYMSKVTHLPLAQLATRLILGQSLATLGLVPGLLTPAPQQIAIKAPVFSFNKLPQSPVVLSPEMKSTGETLGIGPTFTTAWHAAMADSYHLETWQTVDGLITDIATVAQPAVQELFAANHLTVTTIANTTDWPAKTKAVGFTLNDQPDNPAAIAALNHGQPLITAIDTLKTLLASTTTMVSE |
| L.plantarum (SK151) | >CJIJOCHA_01235 Carbamoyl-phosphate synthase large chain (carB) |
| L.plantarum (SK151) | MNNQALQKVLIIGAGHNDIGREGQHDAAVTQIGAAIRRLGIAVVLVDNNAYSVAAENRFADKVYLEPLTVAALSKIIETEQPDGLIPSLGGVQTVTLIQGLIRNNVLKNNHVKLLQWDQDVVDMIVNPALMSNRLKDMGEPVIASQVVASTTEAMAVVRHVGFPVIVKSVAPRIEASRQLCEDEDELQRALAMGFKVSGTKQCIVEQSIVGYKELEFVAVRDQKDTALLVSGMENFDPVGIHSADSIVFAPTQTITDQEYQQFRSATLKIMRKLHLTGSCHVQFALDPTTQNYYVIKVTPYFDRTMALAARATEYPLAQVVGNLMVGISLAEVKLPGAYRKQTALIEPVLDHIVCRIPVWPFNVLKKVSHQLDTVTESTGSVIGIGRSTEEALLKGLRSTDESRYHVIANRPQNYSEGELIQRLIHPQAGRMLILLEALNRGYQIDELAELTKIDAFYFYKLSHILEIERALRERPFDVHALGRAKYFGFTDEDAAAAWQTDVAKVRNFALENEIQPTFKEVEPTAGEFTEQTSTYYSTYEFDNESQPSTGRRVVVIGTGANRIGTNHGNDYLVSQLLFYLKKADYEPVLINANPNSVAMTPRLAKKRYVEPKQYSDILNVLAIEKPLIVFTTYHDYRLGQKLVQDGYQVIQINNSLPREQIKTNDSPAVEVITDGTDVTIFGVSEIIAGNNLRGNVRGAVQAFPIRDKAENQAITALHAEIKQRVLAMGAPGLYTVRLAIADTALQLATIEPMSIATMALISKASGSSVTRLLLHVKLGEALTKVMYDYPDVNRLKPVYVQANTFPYNHLQIYDEVEATGEPGHATGTVIAHGDTLKAALTALNQGNEDTDF |
| L.plantarum (SK151) | >CJIJOCHA_01518 Pyruvate carboxylase (pyc) |
| L.plantarum (SK151) | MKKVLIANRGEIATRVIRACHELGLQTVAIYAKEDEFSVHRFKADEAYLVGEGKAPIAAYLDIEDIIRIAKENHVDAIHPGYGFLSENATFARRIAEEGMTFVGPKPEHLEMFGDKITAKRVARDAGVQTIPSTLHPVTSLNEALQFTQQYGYPIMIKAAMGGGGRGMRIVHEASELQEAFDRARSEAMQSFGDDEIYLEKFIANPKHIEVQILADAHGNVMHLFERDCSVQRRNQKVIEFAPAVALPIELRQKICNAAVDLMKSVHYLNAATVEFLVEGDQFYFMEVNPRVQVEHTVTEMITEIDIVHAQLKIAMGGDLFEDLRLPHQDALTYKGAAIQCRITTEDPANDFMPDTGRIETYRSPGGNGVRLDAGNTYSGAIVTPYFDSLLVKACVAARNFKAAVHKMRRVLVEFDIRGVKTNIPFMLNVIDNPTFQAGEAGTRFIDQTPTLFKFPDDTQREDKMLKYIGNVTVNGFADVAQHSKKYYPDVEFKDDFQPIAPDIVTAKDVLDQQGVQGLQQWLLAQKQVLLTDTTMRDAHQSLFATRMRTKDMLAVAAASQKALPQLFSYEMWGGATFDVAYRFLNENPWNRLKELRQAMPRTLFQMLFRGSNAVGYQNYPDNVIREFILEAAKSGIDVFRIFDSLNWIPQMEKSIQAVKETGKIAEATICYTGDIMDPDRQKYNLDYYRQLALDLQSTGADIIAIKDMAGVLKPEAAYELVSTLKDALSVPVHLHTHDTTGNGIFTYARAVEAGVDVVDVAASALSGTTSQPSMSSLYYALAHNDRQPDVDINNVEAINRYWQGVRPYYQDFSNGMTGPQTDIYQTQMPGGQYSNLQQQAKAVGLGDRWEEVKTMYATVNDMFGDIIKVTPSSKVVGDMALFMMENHLTPDDVYNQGTKLDFPASVINFFAGNLGQPVGGFPKKLQQIVLKGQPALTVRPGSLAKPADFAAVKAELSAKLGHEASHQEVLSYILYPKVFMDYDASHKRYGHVSLLDTPTFFQGMRLGETVNIELAKGKTMIVKLNQISDPDVDGVRTLYFSINGQNQEIMVKDNAIHQSATSTRKAEPTNEDEVGATMSGSVLKLLVKKGQTVKKGEPLLVTEAMKMETTIQAPEDGVIEHIYVNAGDVIQTDDLLLEIAPQ |
| L.plantarum (SK151) | >CJIJOCHA_01082 Acetyl-coenzyme A carboxylase carboxyl transferase subunit alpha (accA) |
| L.plantarum (SK151) | MSKVTAYEQVQAARDASKISIQTLIDGLTEDFFDCHGDRQRADDPAVIAGLATIARRPVTIIGIQKGQNLAENQARHFGCATPSGYRKALRLMRQAEQLRQPVVTLINTPGAYPGVDAEYEGQGRAIADCLLAGLQLRVPFLSLIVGEGGSGGALALACGDQVWMLANSTYSVLSPEGYATILWKESQRAAEAAEKMRLTPTELLADGIIDRIIPEVATAADCQPLKTAIDETLTALTAKSVTELVTQRQARYRQF |
| L.plantarum (SK151) | >CJIJOCHA_00183 Acetyl-coenzyme A carboxylase carboxyl transferase subunit alpha (accA1) |
| L.plantarum (SK151) | MDKRLQRLRAADRISAPTIIQALLPTMTQQRGDRLLGQDAALTAGFGRTATGRQLAVLGVDRGVDIASRRRKNGGALTVPGYRTALRVVTHAAKFGWPVVSLINMPGADASPQSERYGQSQAIADLIAATGQLTVPNVVVFLGEGHSGGALAFANANRIIMLDDALFNVASPEAVTAILHGQQTSSDAIDLLPMTASALQSRGLVDLVIDHQDQTLVAAINQAIETQLAALEALPAPDLIEQREAKFQHFLQSWPVN |
| L.plantarum (SK151) | >CJIJOCHA_01078 Biotin carboxyl carrier protein of acetyl-CoA carboxylase (accB) |
| L.plantarum (SK151) | MSMTDTEAMTALINQFNQSSAQVMDVKTTTFELHLNKQAETAETNSARSQPAVTDSAVPVVKPTTEVATQSGTTMVTAPLVGVAYLAADPKQAPFVQVGDRVKSGETLCVIEAMKMINEVPSPVSGIVKEVLIKNATMVEFDEPLFAIEETTV |
| L.plantarum (SK151) | >CJIJOCHA_00180 hypothetical protein (accB1) |
| L.plantarum (SK151) | MELKDLDRLVEKYARQGYQQIRVKAGDLEIEVTKPALASETVTPVAATPASTVAETEDEVVTLPSPMVGVVHLAPDLKVGQSLTAGQELGQIESMKLFNPLVSPTDGTLTAVLVADDATVEYEQPLFTMRKDR |
| L.plantarum (SK151) | >CJIJOCHA_03059 Biotin carboxyl carrier protein of acetyl-CoA carboxylase (accB3) |
| L.plantarum (SK151) | MELDKIYQLMDKFEQSSLTSFEYRDQDFEIQMGKKGHGKTTVTPQPAPLPNQPVIDPTPVTPTPVSTVSTPVVAEAEPTPVATSMASNPTSVVTPDPEPEPAPIDPKECVTAPVVGIYYPAHSSEEAPYVKLGDHVSVGQQVGLIQAMQMMRPVVAKQAGTVKAFLVKNGEEVNFGQPLIQLIPDTPDDI |
| L.plantarum (SK151) | >CJIJOCHA_01080 Biotin carboxylase (accC) |
| L.plantarum (SK151) | MFKKVLVANRGEIAVQVIRALHEMGIKAVAVYSVADQESLFVHLADEAVCIGASPVNQSYLNMQAIISAANLTGCQAIHPGYGFLSENAEFAKMCADCQLTFIGPRPEVIDQMGDKENARQTMQRLGVPVIPGSPSVLKDVEAAMQAAHTLGYPVMIKAAAGGGGKGIRAVSNDQDLAKAFRTAQQEAQASYDDHRLYLEKIIAPAKHIEVQVLADQQGHVIYLPERDCSLQRNHQKVLEESPCAVITTAERTELGQLVADATKKLGYTNTGTYEFLMDQQHHFYFLEMNTRLQVEHTVTEMVTGIELIKAQVQIAAGMPLKIKQADVKIHGVAMECRLNAEDPYHDFRPQPGKITRLIYPMGTLGVRIDAGVVTGSMIAPFYDSMIAKIIVHGINRKMVSRKMHRCLLELTIDGVQTNRIFLAGLVADSTVGRGTYTTAYIEQDFLKGWLSDAQAQVSSTH |
| L.plantarum (SK151) | >CJIJOCHA_00181 Biotin carboxylase (accC1) |
| L.plantarum (SK151) | MFRKLLIANRGEIAVRIIKACQQLNIQTVAVCSTADRHAGYTQLADEVVCIGPAAASGSYLNAEAILMAAINTHADAIHPGYGFLAENADFAAMCAECGIAWIGPQTEQIKLMGDKARAKDFARQQAVPVLAGQSLQGLAWPDIKHAAAKIGFPLVLKASYGGGGKGIRVLTDVHELHQQLRATRQEAAASFLNDAMYLEQFLTTARHIEVQVLGTGTKLFILGDRDCSLQLHKQKVLEESPASILTETQRRTLYDLSRQLLAGTHYQSLGTIEYLFADGQFYFMEMNTRLQVEHGVTELTTGIDIVAEQIKQAAGVTVDFPEHVGLQPKCTAIEARLTATLPVKQTRIETLSWPADVRVDTDYHQGDRLVTMYDGLIAKLMVTGVNRQQAVRNLQRALAQVTLTGPTTNLTFLQQTVARPAYIHDQYTIHSLARWEASK |
| L.plantarum (SK151) | >CJIJOCHA_01081 Acetyl-coenzyme A carboxylase carboxyl transferase subunit beta (accD) |
| L.plantarum (SK151) | MPKRKFQAPTERQLAVRRDNIPDALLTRCPVCHEDCYTQDLGEFKVCPHCDYGFRLPAWQRVQQLTASFEERDADLSAPVSFDDPAYLKKLQRAKAASHLNESVLTGIGTLATYQFGLGVMETKFMMGSLGAATGEKITRLFETCTTQKLPVVMVTASGGARMQEGARALMQMAKVSTAVANHRKAGLLYITILTDPTTGGVTASFAMQGDIMLSEPRALIGFAGRRVIEQTIQQTPPADFQRAETLLANGWLDQIVPRPALRKTLQRLLTITQGGHQDV |
| L.plantarum (SK151) | >CJIJOCHA_00182 Acetyl-coenzyme A carboxylase carboxyl transferase subunit beta (accD1) |
| L.plantarum (SK151) | MSHYPASGTWQACPKCGRHVHQRQWGTYQQCPYCHYWQRLTTAQRLEQLVDEGSFQPLTMTERPVNQLGFPDYTNKLRRAQRQTGLNEAVVCGTALIEQQPCILAVMDSHFMMGTLNTAVTRRLLHASEQARAQRLPLIIVTASGGARMQEGVYALVGMNLILAELARLAATPLPLITVLTDPTMGGVSASFAFKGDLIIAEAGAKIGFAGARVIQQTLPVKLPADFQTADQLFKNGMVDAVVERPQLRSTLGQALVNYGIGRSAHG |
| L.plantarum (SK151) | >CJIJOCHA_00179 3-oxoacyl-[acyl-carrier-protein] synthase 3 (fabH) |
| L.plantarum (SK151) | MPTYTKLTAMGQYVPARVVDNDELAAIMDTSDEWIQAHTGIKTRHYAMNDENTSDLATQVAQQLLQKSGLAASAIDLILVTTITPDALTPATACLVQANIGADNAFAFDLSAACAGFTFGLATADKFIRSGQYQNVMVISAEVNSKMMDFQDRTAAVFFGDGAGGALLQATDNPDENSLIAEKLESQGNATVIHSGRVRPITEVAATNYPQTDAFYQVGRDVFQFATTVVPEQMRGLIASAQLTPSDLQYVICHQANLRIIEKIAANLALPMTKFPHNVDHYGNTSSAGVAMALANVFDTLTGPVLLTAFGGGLAYGSVLIKK |
| L.plantarum (SK151) | >CJIJOCHA_01073 3-oxoacyl-[acyl-carrier-protein] synthase 3 (fabH) |
| L.plantarum (SK151) | MTTNFSILASAKALPTTKVTNQELTQLMATSDDWIKQRTGIRSRHVATDETTTSLAVSVAQQLLQQSRLAATAIDLIIVATMSPDYLTPATAPQVQAAIGAEKAIAFDINVACAGFVYGMQLVHQYLQPGQTALLIGSETLSRLVDWHDRSTAVLFGDGAGGLLITAKPNTTTGHWLGGHYATFGADGHYLTAGQQPQVNPWSTRTDGADSNRWAFQMNGRRVYDFATKQVPHSIEQALMQARLESSDIKAFVLHQANARIVKSVGQKLNLATEQLPMNIAQYGNTAAASEPILFAEMVAQKQVQRGDKLVFTGFGGGLSVGSVVIEY |
| L.lactis (LAC640) | >NNLFFCON_01266 Glycerol uptake facilitator protein (glpF) |
| L.lactis (LAC640) | MHSEMVQLLGEFLGTFILILLGNGVVSGVVLNKTKATGAGWVAITLGWGSAVMMGVYISGFMSPAHLNPAVTIAMAMIGSFSWSLVLPYIIAQMLGAMVASIILYLMFYPHYAETKNPADILGTFSTGPALRHTGSNLISEIVGTAVLTTGILAFGQYTITQTSGVSPLLVGAIITAIGLSLGATTGYSLNPARDLGPRIMHAILPIKGKGDSDWSYAWIPVLGPIIGGSLGALLFNMVIQFASN |
| L.lactis (LAC640) | >NNLFFCON_00217 Glycerol uptake facilitator protein (glpF) |
| L.lactis (LAC640) | MTLQILGEFLGTFILVLLGNGVVAANVLGKTKSENAGWVTIAIGWGLAVAVAVWVVGFFSPAYLNPALVIGFLVLGKIKIGLAFAFIVAEFLGAMLGAVAVWLHYYPHWEETKDSSLIFASFATAPAIRHSWSNFLGEAFDGALLMILIMAMGHYRLEGGFGPIIVGFLIMAVGFSLGPTTGYAMNPARDLGPRIMHALLPIKNKGTSGWGYAWIPATGSIVGAVIAGLLYQWMLTLH |
| L.lactis (LAC640) | >NNLFFCON_01608 Carbamoyl-phosphate synthase small chain (carA) |
| L.lactis (LAC640) | MSKRLLILEDGTIFEGESLGANLDVTGELVFNTGMTGYQESITDQSYNGQILTFTYPIVGNYGVNRDDYESIHPTCKAVVVHEAARRPSNWRMQMSFDEFLKAKNIPGITGVDTRAITKIVREHGTMKASLVQARDEVEHQMSQLQATVLPTNQVETSSTTTAYPSPNTGRKVVVVDFGLKHSILRELSKRECNLTVVPYNTSAKEILEMEPDGVMLTNGPGDPTDVPEAIEMIKEIQGKIPIFGICLGHQLFSLANGAKTYKMKFGHRGFNHAVREIATGRIDFTSQNHGYAVSSENLPEDLMITHVEINDDSVEGLRHKHFPAFSVQFHPDAAPGPHDASYLFDDFMDLMDNFKK |
| L.lactis (LAC640) | >NNLFFCON_01393 Carbamoyl-phosphate synthase large chain (carB) |
| L.lactis (LAC640) | MPKRNDIKKIMIIGSGPIIIGQAAEFDYAGTQACLALKEEGYEVVLVNSNPATIMTDREIADTVYIEPITLEFVSKILRKERPDALLPTLGGQTGLNMAMELSKTGILEELNVELLGTKLSAIDQAEDRELFKELCERIGEPLCASDIATTVDEAVEIADKIGYPIIVRPAFTMGGTGGGICDTEEELREIVANGLKLSPVTQCLIEESIAGYKEIEYEVMRDSADNAIVVCNMENFDPVGVHTGDSIVFAPSQTLSDNEYQMLRDASLNIIRALKIEGGCNVQLALDPHSYEYRVIEVNPRVSRSSALASKATGYPIAKMSAKIAIGMTLDEIINPVTNKTYAMFEPALDYVVAKIARFPFDKFENGDRHLGTQMKATGEVMAIGRNIEESLLKAVRSLEIGVFHNDLQEAQEADDEILYEKMVKTQDDRLFYVSEAIRRGIPIEEIADLTKIDIFFLDKLLHIVEIEDQLKVNIFEPELLKTAKKNGFSDRQIAKLWNVSPEEVRRRRQENQIIPVYKMVDTCAAEFESSTPYFYSTYEWENESKRSSKEKIIVLGSGPIRIGQGVEFDYATVHCVKALQALGKEAIVINSNPETVSTDFSISDKLYFEPLTFEDVMNIIDLEQPEGVIVQFGGQTAINLAEPLSKAGVKILGTQVEDLDRAEDRDLFEKALQDLEIPQPPGATATNEEEAVANANKIGYPVLIRPSFVLGGRAMEIINNEKDLRDYMNRAVKASPEHPVLVDSYLQGRECEVDAICDGTEVLLPGIMEHIERAGVHSGDSMAVYPPQTFSQEIIDTIVDYTKRLAIGLNCIGMMNIQFVIFEEQVYVIEVNPRASRTVPFLSKVTNIPMAQLATQMILGKNLKDLGYTEGLAETPDMVHVKAPVFSFTKLAKVDSLLGPEMKSTGEAMGSDVTLEKALYKSFEAAKLHMADYGSVLFTVADEDKEETLELAKDFAEIGYSLVATQGTAAFFKENGLYVREVEKLAGGEDEEGTLVEDIRHGRVQAVVNTMGNTRASLTTATDGFRIRQEAISRGIPLFTSLDTVAAILKVMQSRSFTTKNI |
| L.lactis (LAC640) | >NNLFFCON_00581 Pyruvate carboxylase (pyc) |
| L.lactis (LAC640) | MKKLLVANRGEIAVRVFRACNELGLSTVAVYAREDEYSVHRFKADESYLIGQGKKPIDAYLDIDDIIRVALESGADAIHPGYGLLSENLEFATKVRAAGLVFVGPELHHLDIFGDKIKAKAAADEAKVPGIPGTNGAVDIDGALEFAKTYGYPVMIKAALGGGGRGMRVARNDAEMHDGYARAKSEAIGAFGSGEIYVEKYIENPKHIEVQILGDNHGNIIHLHERDCSVQRRNQKVIEIAPAVGLSLDFRNEICEAAVKLCKNVGYVNAGTVEFLVKDDKFYFIEVNPRVQVEHTITELITGVDIVQAQILIAQGKDLHREIGLPAQSEIPLLGSAIQCRITTEDPQNGFLPDTGKIDTYRSPGGFGVRLDVGNAYAGYEVTPYFDSLLVKVCTFANEFSDSVRKMDRVLHEFRIRGVKTNIPFLINVIANENFTSGQATTTFIDNTPSLFNFPHLRDRGTKTLHYLSMITVNGFPGIENTEKRHFEEPRQPLLNLEKKKTAKNILDEQGADAVVDYVKNTKEVLLTDTTLRDAHQSLLATRLRLQDMKGIAQAIDQGLPELFSAEMWGGATFDVAYRFLNESPWYRLRKLRKLMPNTMFQMLFRGSNAVGYQNYPDNVIEEFIRVAAHEGIDVFRIFDSLNWLPQMEKSIQAVRDNGKIAEATICYTGDILDPSRPKYNIQYYKDLAKELEATGAHILAVKDMAGLLKPQAAYRLISELKDTVDLPIHLHTHDTSGNGIITYSGATQAGVDIIDVATASLAGGTSQPSMQSIYYALEHGPRHASINVKNAEQIDHYWEDVRKYYAPFEAGITSPQTEVYMHEMPGGQYTNLKSQAAAVGLGHRFDEIKQMYRKVNMMFGDIIKVTPSSKVVGDMALFMIQNDLTEEDVYTRGNELNFPESVVSFFRGDLGQPVGGFPEELQKIIVKDKAVITDRPGLHAEKVDFETVKADLEQKIGYEPGDHEVISYIMYPQVFLDYQKMQREFGAVTLLDTPTFLHGMRLNEKIEVQIEKGKTLSIRLDEIGEPDLAGNRVLFFNLNGQRREVVINDQSVQTQVVAKRKAETGNPNQIGATMPGSVLEILVKAGDKVQKGQALMVTEAMKMETTIEAPFDGEIVDLHVVKGEAIQTQDLLIEIN |
| L.lactis (LAC640) | >NNLFFCON_00742 Acetyl-coenzyme A carboxylase carboxyl transferase subunit alpha (accA) |
| L.lactis (LAC640) | MSETIDSLTGEVSQKNIADIINEARAADRISARDIIDKVFTDFFELHGDRQLTDDEAIVGGIARFNNQAVTVIGIQKGRNLAENLATNFGQPSPNGYRKALRLMKQAEKFGRPVITFINTAGAYPGIEAEERGQGEAIARNLLEMSDLKVPIIAFITGEGGSGGALALALANKVYILENAVYSVLSPEGFATILWKDGSRRDEAAELMKITAPHLLEMGLVDAIISEENLIENLKRQLTESLGELSLLTEIELAEQRYERFRKY |
| L.lactis (LAC640) | >NNLFFCON_00738 Biotin carboxyl carrier protein of acetyl-CoA carboxylase (accB) |
| L.lactis (LAC640) | MNISEVKDLMNQFNGSTLREFSWKNADGELSFSKNEGQALVGAQSAAPTAPAFTAQQATPVAESTEVPVASEAAVEAEGEAVTSPLVGVAYLKPSPDKAEFAKVGDSVKKGQTLLIIEAMKVMNEIPAPKDGVITEIMVSGEDVVEFGQDLMRIK |
| L.lactis (LAC640) | >NNLFFCON_00740 Biotin carboxylase (accC) |
| L.lactis (LAC640) | MFNKILIANRGEIAVRIIRAARELGVGTVAIYSTADRDALHVHLADEAICVGPARSTESYLNMNNILSAAVATGAQAIHPGFGFLSENSKFARLCEEMNIKFIGPSAKVMDMMGDKINARAEMIKAGVPVTPGSEGEVHTTDEAVKIAEEIGYPIMLKASAGGGGKGIRKVNNVEELVPAFEAAQAEAQAAFGNGAMFIERMIFPARHIEVQILGDSQGNVIHLGERDCSLQRNNQKVLEESPSVAIGHTLRQEITAAAVKAAQHVSYENAGTIEFLLDESSGKFYFMEMNTRVQVEHPVTEFVSGVDIVKEQIRVANGNELSVKQEDVTFSGHAIECRINAENPKFNFAPSPGKITNLFLPSGGVGLRVDSAMYSGYSIPPYYDSMIAKVIVHGENRFEALMKMQRALLEFDVEGVITNVDFQLELISDPHVVAGDYDTSFLGNVFLPEYQKED |
| L.lactis (LAC640) | >NNLFFCON_00741 Acetyl-coenzyme A carboxylase carboxyl transferase subunit beta (accD) |
| L.lactis (LAC640) | MALFQKKKYIKINPNRSIIEKQAEQPEVPDELFAKCPACKHTIYQKDLGKNKVCPNCDYNFRITAKERLAIVADKDSFVEMFTGIESKNPLDFPGYPEKLAATKARTGLDEAVMTGTATIKGQKTALAIMDSTFIMASMGTVVGEKLTRLFEYATTEKLPIIVFTASGGARMQEGIMSLMQMAKTSAAVKRHSNAGLFYITVLTDPTTGGVTASFASLGDIILAEPQSLIGFAGRRVIEQTVRQTLPDDFQKAEFLLNHGFVDAIVKRTELRQKLALLLELHTEVENV |
| L.lactis (LAC640) | >NNLFFCON_00733 3-oxoacyl-[acyl-carrier-protein] synthase 3 (fabH) |
| L.lactis (LAC640) | MTFAKITQVAHYVPENVVSNDDLSKIMDTNDEWIYSRTGIKNRHISTGENTSDLAAKVAKQLISDSNLSPETIDFIIVATVTPDSLMPSTAARVQAQVGAVNAFAYDLTAACSGFVFALSTAEKLISSGAYQRGLVIGAEVFSKVIDWSDRSTAVLFGDGAAGVLIEAGGSQPLIIAEKMQTDGSRGNSLLSSYADIQTPFASVSYESSNLSMEGRAIFDFAVRDVPKNIQATLEKANLSAEEVDYYLLHQANSRILDKMAKKLGVTRQKFLQNMQEYGNTSAASIPILLSESVKNGIFSLDGQTKVVLTGFGGGLTWGTAIINL |
| L.brevis (NPS_QW_145) | >HEBPGAIP_02242 Glycerol facilitator-aquaporin gla (glpF) |
| L.brevis (NPS_QW_145) | MVALGNGSVANVELKGTKGYHGGWVLIGFGYGIGVMIPALMFATVSGAQINPAMTLAMAVTGNFPWGEVAPYIVAQVLGAIIGQLTLVLAYKPYYDKTTDAEAILGTFSTIDAADSKLNGFINEFIGTFLLVLGAISMTADKLDPRADFIGLGFLVMCLVISFGGPTGPALNPARDIGPRLLHAIWPFEHKGSSQFGYSWVPIVAPIAGAIAAAVLYQGVFQ |
| L.brevis (NPS_QW_145) | >HEBPGAIP_01972 Glycerol uptake facilitator protein (glpF) |
| L.brevis (NPS_QW_145) | MSGFIGEFFGTMILIVLGAGTGASANLKKTYGHGSNWTFISIAWGLAVTMGVYVAGALGSDGHLNPAVTIGFAVFGFFPWSSVVPYLLGQFLGAFVGAVIVIIQFYPHFKATPSVEEGNTVGIFATRPAIANPLFNFLSEVIATWAFIFILLNLGDFTQGLKPFIVGMLIAIIGMGLGTTTGFALNPARDWGPRLAHSLMPIPHKGSSEWSYAWVPMCGPIVGGILAAGLQVLVK |
| L.brevis (NPS_QW_145) | >HEBPGAIP_00180 Carbonic anhydrase (cah) |
| L.brevis (NPS_QW_145) | MSILNYAEQASWSNGFGSQQSPIDLRQAKPTSVTNFVVTDPWIADREIDDQVTIQLAGHGTTRINEMTWHFVQAHIHVPAEHIATEETVAELHFVHQSVIGALCVVAVLVPIGKANPIIDGVLDHFQPHATESIDCDLTGLLPNRGTVFQYLGSLTTPPLTEGVTWYVIEQTDITISSEQLKRYKQLFGPNNRQVQATNQRSVLRGSGTFTK |
| L.brevis (NPS_QW_145) | >HEBPGAIP_01462 Carbamoyl-phosphate synthase small chain (carA) |
| L.brevis (NPS_QW_145) | MAKRYLILEDGSAYAGEGFGAGATTSGEVISNLNLLGYQETITDQIYHNQIIVFAQPAIGNVGINHDSYESILPTAKGMVVRDVTNISTNRLSRLSLDEFLQQHNIPGISGIDTRHLIRKLRQLDGPMKGSIVDVADAHAFDQLNATVLTNRQVDQVATPKPYPNPDTGKNVVVIDFGLKHGILRQLSERRCNVTVLPWTASAQDVLNLDPDGVLLSTGPGSPLDLGDAVLEMIRTVQAEIPLFAIGLGHELFALANGAELKMLPVEYHGSGHPIRRIVTNDIIYATQGQGYAVVAKSIDRDRLITTYVDLINGTVQGLRHRDFPAFSVQFFADGAPGPHESRDLFDEFMEAMTAREG |
| L.brevis (NPS_QW_145) | >HEBPGAIP_01365 Acetyl-coenzyme A carboxylase carboxyl transferase subunit alpha (accA) |
| L.brevis (NPS_QW_145) | MDKSAYQTVQAARSQDKITTEALIDGLVTDFFECHGDRVDGDDPTIIGGIGRLNGQPITVIGTRKGRNLTENVARHFGSPMPQGYRKVQRLLQQAEKFGRPVLTLINTPGAYPDVASEFHGQGEAIAQCLYQGMQLKVPYISLIFGEGGSGGALALACGDRVYMTVDSIYSVLSPEGYASIMWKDASRVREAAAALALTPQDLKREKVIDDILPEVHTAAELSQLTDFLHEQFKILGKLPISTLIDQRQTRFDQY |
| L.brevis (NPS_QW_145) | >HEBPGAIP_01361 Biotin carboxyl carrier protein of acetyl-CoA carboxylase (accB) |
| L.brevis (NPS_QW_145) | MKNEDIEHLLEKFDHSSLKDFHLVQDDFQLSLSKREDTNVPTPATIDQPTPEPAGETAKESVEPTITAPLVGVVYLAPAPEKPVFKQIGDHVEKGDVVCVVEAMKMINEVKSDLTGTLTKVLVTDGSMVEYDEPLLQIKPD |
| L.brevis (NPS_QW_145) | >HEBPGAIP_01363 2-oxoglutarate carboxylase small subunit (accC) |
| L.brevis (NPS_QW_145) | MMKVSDEMFTKVLVANRGEIAVQIIRALHDMQISAVAVYSQADRESLFVKLADEAVCIGGPQPADSYLNMTQIISTAKLLGCQAIHPGYGFLSENATFARLCQQCQITFIGPNADLIDLMGDKAHARAAMQANQVPVIPGSDGVLDSADQAVQIAQEIGYPVLLKSAAGGGGKGIREVQTAAELPAAFEQVQQEAKLSYGDAAVYLEKCIGQAKHIEMQVIADNFGEVVYLPERDCSLQRHHQKVIEESPCGMIDAKQRQWLGQTVARATRKLGYTNTGTFEFLMDKDHHFYFIEMNTRLQVEHTITEAVTGIELVKAQIRVAANQPLGFTQADVKLNGYAIECRLNAEDPQHQFAPCPGKITALYLPAGSLGVRIDSGVIAGSVISPYYDAMIAKLIVSSQQRTTALAKMDRILGEMQIEGIKTNRQFLRQLLTQRAVQAGTFTTTFIAEMRQKGDEADVTSEVQNAQSEEP |
| L.brevis (NPS_QW_145) | >HEBPGAIP_01364 Acetyl-coenzyme A carboxylase carboxyl transferase subunit beta (accD) |
| L.brevis (NPS_QW_145) | MLPAKFKTPSQKSLNRRMASIPDGLVRRCPVCHTTFLTDHWEPTRLCPACGYGFRLTAMQRIKLTMDTFQETNAQLTVPDRYTDAAYQAKIARGRQLTGLNESVLTGFGTIDHQSTAIGVMDAFFVMGSLGTATGEKITRLFDEATAKQLPVILFTASGGARMQEGIHSLMQMAKVSAAVARHSQAGLLYIVVLCDPTTGGVTASFAMQGDLILAEPHALVGFAGRRVIEQTIHQTPPADFQRAETVLQHGFIDAIVARPQLKQRLADLLRLHKGES |
| L.brevis (NPS_QW_145) | >HEBPGAIP_01356 3-oxoacyl-[acyl-carrier-protein] synthase 3 (fabH) |
| L.brevis (NPS_QW_145) | MKFENFNILATAGYTPERVVPNSELTTMMTTSDEWIVQRTGIHQRHVVTAERTSDLCTRVAQQLLQRSGLQATDIDYIVVATMSPDYQTPAVSAQVQGNIGATKAVALDVNAACSGFVYGLQVVHRLLMGDSPKTALLIGGETLSRLVDWQDRSTAVLFGDGAGGVVLTSALAQDGAFIAADYRTMGEQGRYLTAGANGVDSPFASDPQPVLPFFKMNGRRVYNFAIKQVPASLRRVLDQGHLAVTDVDYFILHQANQRIIERIAEDLTGSMAQFPVNIGQYGNTAAASEPLLLNQLVTSHVIKRGDVLALSGFGGGLTIGTMILRY |
| L.buchneri (ATCC4005) | >PCFFGLNM_01866 Glycerol uptake facilitator protein (glpF) |
| L.buchneri (ATCC4005) | MNGFIGELFGTYVLIVLGTGACAGANLKQTYAKGSNWTFISVAWGMAVTMGVYVASSLGSLGHLNPAVTIPYAIFGLFPWNQVVPYLLGQFLGAFLGAATIMMIFYPHFRTTTVDNGNTVGIFATRPAIYSPVSNYLSEVVATFVFVFALLNLGDFTQGLKPLVVGLLIMVVGLGVGTTTGFALNPARDWGPRFAYSVIPVPNRGSGEWYYSWVPMFGPLTGGIIAAGLQVMVK |
| L.buchneri (ATCC4005) | >PCFFGLNM_01780 Carbonic anhydrase (cah) |
| L.buchneri (ATCC4005) | MLDYKNQAKWPNGFGSQQSPIEIKTAQTLKSKEPLDYHVVSDYQLHQEVDDGTTVRLTGEGAARIFNREYHFVQVHFHAPSEHVIDGKHYPLEIHLVHQNDIGQLVVVALLVSAGELDDEKLQQIMTTYENGKTEPVDLNISDWAPWYPVGFHYLGSLTTPPLTEGVEWLVLTNPEFTISDRQIDWFHQHFGDDNRDPQPLNDRQVELYK |
| L.buchneri (ATCC4005) | >PCFFGLNM_00966 Carbamoyl-phosphate synthase arginine-specific small chain (carA) |
| L.buchneri (ATCC4005) | MKKYLTLENGDVFVGEACGDLGAEIDGELVFTTSMTGYQETLTDPSYKHQIISFTYPLIGNYGISLGTNQSDTVQAAAVVVKEMTDQVFHYQSVISLDAFLKQSKVPAISGIDTRQLTKIIRRYGTMKASLTNHPIVDQPKADRSASIIKSATLSAGDTKDVEHVVVVDFGVKDNIVKSLQRPGVDVTVVTPESTFEDIANLHPTGILLSNGPGDPTDYSDFVPVIQKLQDNFPLFGICLGHQLLALANGATTYKMTFGHRGINHPVKNLITGDIYITSQNHGYAVDVDSVKDTPLEVTALEVNDKTCEGLRYPGRPVFSVQYHPEAAPGPHDATKLFDDFIEIMHVNGGVH |
| L.buchneri (ATCC4005) | >PCFFGLNM_02353 Carbamoyl-phosphate synthase small chain (carA) |
| L.buchneri (ATCC4005) | MTERYLILEDGSAYAGKAFGSLATTTGEIVANSTMNGYQEIISNQIYHNQIIVFTQSSIGNTGLDQNAYESILPSAKGVIVREYENLATDHFKRISLDKYLKQHKIPGIYDVDTREIRHHLQKRGNMKASIVDVADDHAFDQLHATVLTNQQVAQVATPKPFPNPDEGANVVVVDFGLKSGILRELSHRDCNVTVVPWNFSAAQILDLDPDGIVLSTGPGSPENLPKSVLDMITEVQSQAPLFAMGLGHELFALANGAKVSQLTVEHHGSNHPIREIITNEIFFSGQEQGYAVDPKSIDHAKLFITHIDLVNGTVQGLRHRDYPAFSVQFSPDGAPGPKDSVGLFDDFMEIMARRRDSNDDRY |
| L.buchneri (ATCC4005) | >PCFFGLNM_00967 Carbamoyl-phosphate synthase arginine-specific large chain (carB) |
| L.buchneri (ATCC4005) | MPKREDIKKVLIIGSGPIVIGQAAEFDYAGSQACLSLREEGYSVILVNSNPATIMTDDEIADKVYLEPLTVPSLKKIINKEHPDAILPTLGGQTGLNLAVELSKDGILDELGIELLGTSLQTINEAEDREKFKDLMEELYQPIPDSKTVYDLQDGLDFAHKIDYPVIIRPAYTLGGTGGGIAHNDDEMTTILNRGLTMSPSTECLVEKSIAGFKEIEFEVMRDHHGSSIIVTGMENFDPVGIHTGDSIVFAPTQTLTDKEYQRLRDASLTIVNALKIEGGCNVQLAQDPNSEAYYVIEVNPRVSRSSALASKATGYPIAKIAAKIAVGLNLDEIMNPITQTTFAMFEPALDYVVAKIPRFAFDKFTNADRKLGTQMKATGEVMAIGSTIEESLLKAVQSLELDQQAQTDLIPAYTRNMSMGDLLEKIKTPTDYRLFEIFAAIGKGATIQQINRSTQIDMYFLSKLENIIKMQQQMTDGLLSADEVLKARKLSFNNTMIQAIHHATDDELTKLDKMSDQHLVYKMVDTCAAEFESTTPYYYSTVGNENESKPLGNSIVVIGAGPIRIGQGVEFDYATVHCVKAIQAAGYNAIIINNNPETVSTDFSISDKLYFEPLTIDSVMNIINLEQPIGVIVDFGGQTAINLTEGLTKHGVSILGTSLHGIEQTEDRHQFEDLLIDQNIAHPKGATATDAPEALEIANHLGYPVLVRPSFVLGGKGMAVVHNDDELNEYLIPALKNSHGEPILIDQYIPGTECEVDILSDGNDVFVPGIMEHLEGAGIHSGDSIAMYPPQTLTDDQKEKIVAIATKIGKQVHAVGMMNIQFIVADEVYIIEVNPRASRTVPFMSKIVKLHLAQLATQLILGKSLAEIGLKPGLHPEPTKVYVKAPVFSFAKLPGAPTALSPEMKSTGEDIGAGGNLQEALHNALFDSYHIDTNHLTGDVLLSAFDANNASLVEQLKGSGFGIQTYHEGMQWPSNLAFALSSEDETTDQKQLVAKALSHQVPVFTAQDTVMGVFQPQLIK |
| L.buchneri (ATCC4005) | >PCFFGLNM_00486 Acetyl-coenzyme A carboxylase carboxyl transferase subunit alpha (accA) |
| L.buchneri (ATCC4005) | MADKTAYQRVKAARRPDKITTAELINHLTSDFFEQYGDRLEADDPAIIGGIGLLNDKPITVVGIQKGSDTDENIARHFGSPTPQGYRKALRLMKQAEKFHRPILALVNTPGAWPDVESEYHGQGSAVAQNIIQGMQLRVPYITIIVGEGGSGGALALSCGDRVFMFEDSIYSVLSPEGYASIMWKDSSKVKEAAEELKLTPEALLADGIIDKIIHEYQPGEDLSALRDFLDQEFSQLAKLPIDDLVDRRNQRYRNF |
| L.buchneri (ATCC4005) | >PCFFGLNM_00490 Biotin carboxyl carrier protein of acetyl-CoA carboxylase (accB) |
| L.buchneri (ATCC4005) | MDEKEIERLLDKFDRSSLKNFELAQDDFKLKFSKREGDDHAVVEDSAQAASASTNGLGHGQNSQPTTVEQPETPVAQNVAEIKAPLVGVVYFAPSPDKPAFKKQGDHVEKGDVVCVIEAMKMINEVKSDVTGTISNILVEDGSMVEYDQPIFQVTKG |
| L.buchneri (ATCC4005) | >PCFFGLNM_00488 Biotin carboxylase (accC) |
| L.buchneri (ATCC4005) | MFKKVLVANRGEIAVQIIRALHDMGITAVAVYSSADKDALFVHLADEAICIGGPQPSESYLNMAQIISAANLTGCEAIHPGYGFLSENAEFAELCETCHIKFIGPSHELIALMGDKANAREAMKKAGVPVIPGSDGVVNTVAQAEQVADQIGFPVLLKSAAGGGGKGIREVDRPEELKNAFEQTQQEARISYDDEDIYVEKLIRQAKHVEMQVIADNFGHVVYLPERDCSLQRNHQKVIEESPCVLISAEERRHLGEIVANATLKLGYTNTGTYEFLMDDEHHFYFMEMNTRLQVEHTITEEVTGIELVKGQLAVAAGEELPFTQADAAVKGHALECRLNAEDPTNNFAPQPGQINHLFFPAGSLGVRIDSGVTNGSFISPFYDSMIAKVIVHLNDRDTVISKMHRILGELEIDGIRTNQSFLDYLIGTDKFHQGIYSTSYIQNEVLSDKEGFHATQSV |
| L.buchneri (ATCC4005) | >PCFFGLNM_00487 Acetyl-coenzyme A carboxylase carboxyl transferase subunit beta (accD) |
| L.buchneri (ATCC4005) | MLRSRFNMPSHEELVKRMDAIPDHVLRECPICHATFFSLRAGSTKTCPNCGYGFRITAKRRAKITFDSFDEIDKSMTVPDQYTDEKYLAKVKKAKKVTGINESVLTGIGTLDKQQVGVGIMDPFFVMGSLGSSTGEKITRLFEEATRKKLPVIMFTASGGARMQEGIHSLMQMAKVSGAVAEHSRAGLLYVVVLCDPTTGGVTASFAMDGDVILAEPHALVGFAGRRVIEQTIMQKPPKDFQSAETVMAHGFIDAIVPRKQMKATLSRLISLHTKENVYGR |
| L.buchneri (ATCC4005) | >PCFFGLNM_00495 3-oxoacyl-[acyl-carrier-protein] synthase 3 (fabH) |
| L.buchneri (ATCC4005) | MTFEDFKIIETASSVPQRVVDNDELSSMMDTSDAWITQRTGIKRRHVAVEETTSSLATAVATKLLAKSGLKPTEIDLIVVATMSPDYLTPSVSAMVQGNLGADQAIAFDIDAACSGFVYGLKVVRQMLKADRPMHAILIGAETLSKLLDWHDRSTAVLFGDGAAGVLMTNQSSESGSFISEDLKTLGKLGKHLTAGQVGVKSPFAAPETSYSPFFKMNGHRVYGFAVKNVPESINRALEKANLTIDDIDCFVLHQANERIIEKIADTLGASMNQFPVNINEYGNTSAASEPILLAELVAKQRIKRGDVIALSGFGGGLTVGTMIIKY |
